# Optimal Synergy between Azulenes and Acenes in Azuacenes with 6-7-5 Ring Topology

**DOI:** 10.1021/jacs.4c11186

**Published:** 2025-01-07

**Authors:** Fei Huang, Marcos Díaz-Fernández, José M. Marín-Beloqui, Lingyan Sun, Yong Chen, Shengpei Liu, Yuxiang Wang, Han Zheng, Silu Li, Cheng Zhang, Jingsong You, Juan Casado

**Affiliations:** † Key Laboratory of Green Chemistry and Technology of Ministry of Education, College of Chemistry, Sichuan University, 29 Wangjiang Road, Chengdu 610064, P. R. China; ‡ Department of Physical Chemistry, University of Malaga, Campus de Teatinos s/n, Málaga 29071, Spain

## Abstract

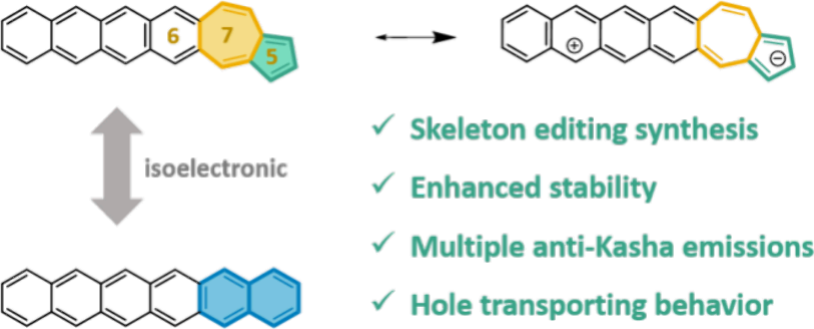

Azuacenes, defined as azulene fused with acenes in a 6-7-5 ring topology and spanning lengths from 3 to 6 rings, have been synthesized using a new skeleton editing and [3 + 2] annulation synthesis protocol as a distinction regarding the procedures to obtain the 6-5-7 isomers. Comprehensive studies on ground-state and excited-state spectroscopy, electrochemical properties, chemical stability, and solid-state structure have been conducted to compare these azuacenes with acenes. For the same number of rings, we found that azuacenes improve the chemical stability of acenes (i.e., smaller diradical character) and their photophysical properties (i.e., anti-Kasha emissions and modulation of the energy and strength of the visible bands) but they reduce the transport features compared to those of acenes. Compared with azulene, azuacenes improve the performance of azulene in terms of electrical properties. Overall, the fusion of known polycyclic compounds, such as acene and azulene, produces new isomeric hybrid compounds with enhanced properties. Here, the resulting compounds turn out to conserve most of the unique properties of the two building blocks that we associate with the facility of π-delocalization of the positive charge of the azulene zwitterion over the acene fragment.

## Introduction

**Figure 1:**
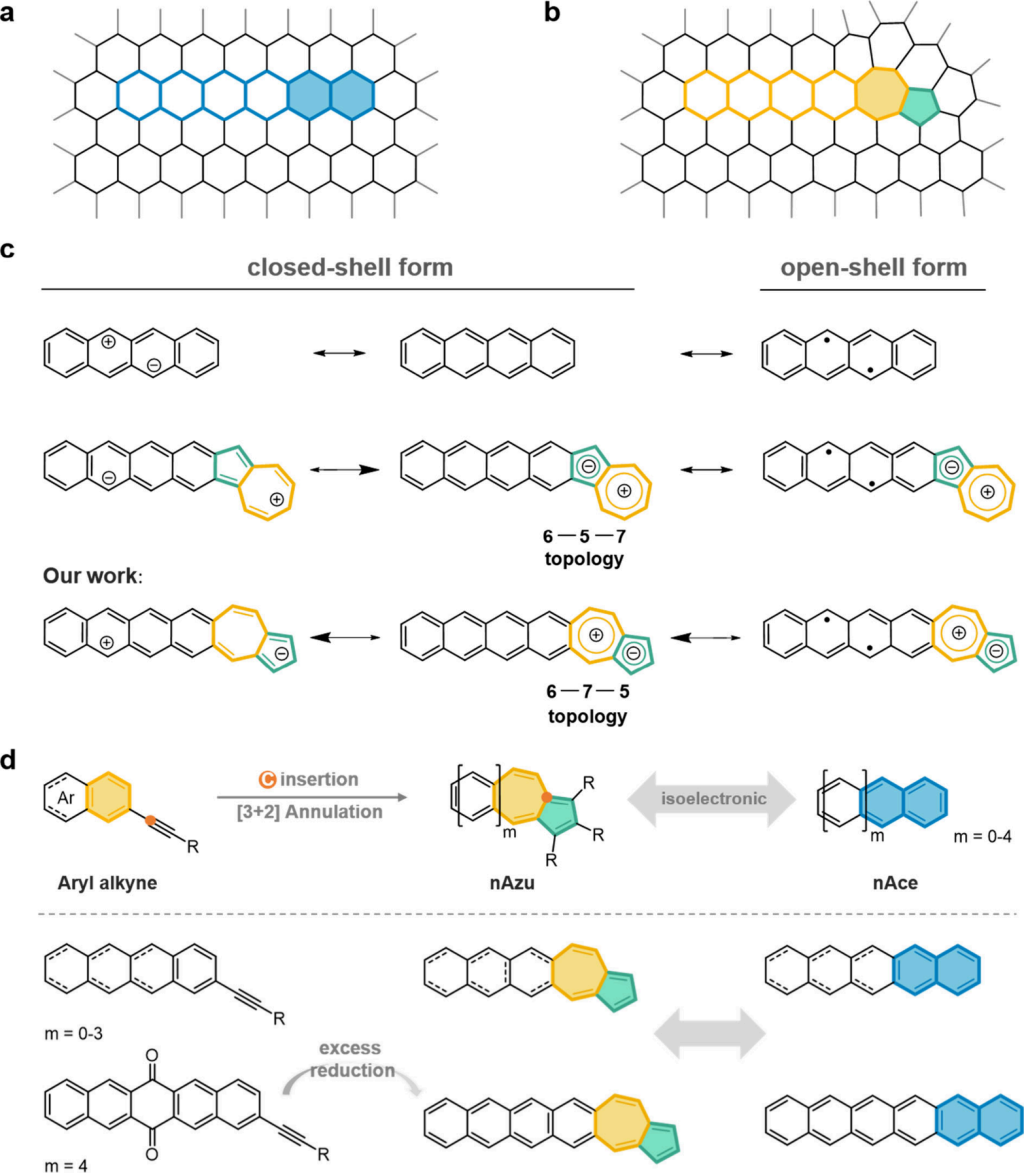
(a, b) Schematic illustration of GNRs (a) without and (b) with structural defects. (c) Resonant forms contributing to the ground-state electronic structure of **nAzu** versus acenes and other azulene-acene hybrids. (d) Synthesis strategy of skeleton editing to generate **nAzu**s and their isoelectronic acenes (**nAces**, *n* = 2–6).

Graphene nanoribbons (GNRs) and nanographenes (NGs) based on benzenoid rings possess enormous potential as next-generation semiconductor materials due to their tunable band gaps and attractive electronic properties.^1^ The incorporation of nonbenzenoid rings into GNRs/NGs breaks down the ideal hexagonal lattice contributing to the electronic property modulation as well as to important aspects such as solubility, stability, and reactivity;^2^ thus, GNRs and NGs featuring contiguous ring size defects and multiple fused nonbenzenoid rings have garnered particular interest (Figure 1a,b).^3^ Monodimensional NGs made by atomically precise polycyclic fused aromatic hydrocarbons (PAHs) or acenes have emerged with a dual purpose: as representative models of corresponding GNRs and as valuable materials for organic optoelectronics.^4^ One can start by mentioning napthalene and anthracene,^5^ wherein singlet exciton fission for photovoltaics was first discovered, and continue with derivatives of tetraphenyl tetracene (rubrene)^6^ and pentacene,^7^ which are the benchmark for organic semiconductors in organic field effect transistors (OFETs). However, acenes longer than pentacene suffer from chemical reactivity, by which these are poorly stable under ambient conditions. Part of this chemical reactivity of long acenes is associated with the emergence of diradical character which promotes intermolecular [2 + 2] dimerization/polymerization and reactions with ambient oxygen as the main channels of molecular degradation.^8^ Long acenes up to undecacene and dodecacene are nowadays studied at low temperatures and in protective inert gas matrixes and have been more recently prepared by on-surface synthesis from suitable ambient stable precursors.^9^

A rather unexplored mode of exploiting the properties of acenes is by structural isomerization of the benzenoid rings into nonbenzenoid 5/7 rings arrays, exemplified by the well-known case of azulene as the electronic isomer of naphthalene. From naphthalene to the nonbenzenoid azulene, the electronic properties are dramatically modified in such a way that azulene features the appearance of visible electronic absorptions (i.e., in the blue) and challenging anti-Kasha emission behavior.^10^ The discovery of new molecules with anti-Kasha activity is of great interest in organic electronics since they expand the spectral range of emissions, allowing several emissions (different colors) to be shown with a single material. Molecular hybrids putting together acenes and azulene in one-dimensional ring fused arrays have been prepared by Chi et al.^11^ using the connection to the benzenoid core through the five-membered ring, thus with a 6-5-7 topology of fusion according to the sequence of fused rings (Figure 1c). These 6-5-7 azulene and acene, or azuacenes, hybrids have been synthesized either in the format of one azulene moiety fused to acenes of different sizes or with two end-capping azulenes. All of these 6-5-7 compounds display improved chemical stability (comparing azulene-acenes and acenes of the same number of fused rings) as well as enriched optical (i.e., visible optical absorptions) and electrochemical behavior. Then, incorporating an azulene moiety via the 5-membered ring produces a molecule that exhibits the properties of an acene while losing all characteristics associated with azulene.

Here, we now posit that the preparation of azulene-acene isomers following the inversed topology, or with 6-7-5 pattern (Figure 1c), of connection between the benzenoid and nonbenzenoid rings represents the optimal way to control and design the electronic and chemical properties of these molecular hybrids. This statement is based on the consideration of the dipolar zwitterionic electronic shape of azulene which bears the positive charge in the seven-membered ring and the negative charge in the five-membered ring. π-Conjugated molecules, given the large number of electrons, always prefer to stabilize positive charges rather than anions, and thus most of their described stable radical charged species are cations and dications. Hence, the best choice to synergistically improve the properties of acene and azulene is by positioning the positive charge of azulene directly connected to the electron-rich acene core, such as in the 6-7-5 topology. However, the synthesis presents challenges due to the chemical inertia of the seven-membered ring compared to the five-membered ring; thus, most of the reported azulene-embedded PAHs were synthesized by the stepwise construction of a five-membered ring followed by cyclization to form the remaining seven-membered ring or vice versa. We now present a bottom-up synthesis approach for the preparation of nonbenzenoid azulene ring embedded acenes, azuleno[5,6-*b*]acenes (hereafter **nAzu**s, **2Azu**-**6Azu**) with the 6-7-5 topology which have been synthesized through simultaneous skeletal editing and [3 + 2] annulation to form the azulene moiety (Figure 1d).^12^ Though we use the same notation, **nAzu**, as used by Chi et al. to denote the 6-7-5 isomers, we clarify that they are isomers in between. To our delight, **5Azu** and **6Azu** are more chemically stable than pentacene and hexacene due to the smaller diradical character and more robust closed-shell form by zwitterionic stabilization. The **nAzu** compounds can be described as superazulenes with tunable anti-Kasha emissions due to their extended dipolar shapes. The dual incorporation of azulene and acene inputs in **nAzu** is further revealed in the conservation of the dimerization mode of crystallization, in the propensity for reversible acid reactions (negative charge protonation), and the extension of the chromatic and photoabsorption features into the near-infrared region. Finally, the fabrication of OFET devices with behavior comparable to that of acene is another distinctive property.

## Results and Discussion

### Synthesis of **nAzu**s

**Figure 2:**
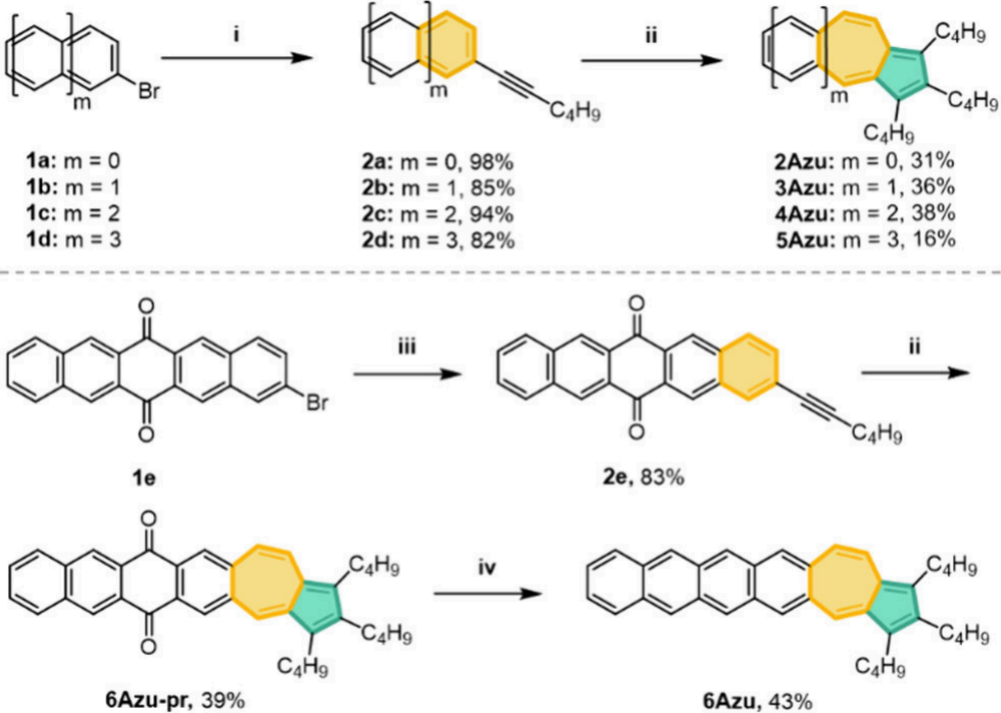
Synthesis routes of 6-7-5 **nAzu**s (*n* = 2–6). (i) 1-Hexyne (1.3 equiv), Pd(PPh_3_)_2_Cl_2_ (5 mol %), CuI (10 mol %), Et_3_N, 80 °C, 48 h. (ii) 5-Decyne (3.0 equiv), Pd(acac)_2_ (20 mol %), B_2_pin_2_ (4.0 equiv), LiI (6.0 equiv), 2-MeTHF, microwave, 150 °C, 4 h. (iii) 1-Hexyne (1.3 equiv), Pd(PPh_3_)_2_Cl_2_ (5 mol %), CuI (10 mol %), piperidine, 100 °C, 24 h. (iv) (1) NaBH_4_ (12.0 equiv), MeOH, THF, r.t., 20 min; (2) SnCl_2_·2H_2_O (2.4 equiv), DCM, AcOH, r.t., 12 h.

The synthesis routes of azuleacenes are described in Figure 2. To address the solubility issue, we strategically selected *n*-butyl as the terminal unit linked to the five-membered ring. Initially, a two-step synthesis route was hypothesized to afford the desired azulene-fused acenes **2Azu**-**6Azu**. Commencing with the palladium-catalyzed Sonogashira coupling of 2-bromoacene (bromobenzene) and 1-hexyne, aryl alkynes (**2**) were successfully obtained with a yield range from 82 to 98%. It is noteworthy that bromobenzene (**1a**), 2-bromonaphthalene (**1b**), and 2-bromoanthracene (**1c**) are commercially available while 2-bromotetracene (**1d**)^13^ was synthesized according to the reported literature. Subsequently, employing the skeleton editing developed by You’s laboratory^12^ enabled us to convert these intermediates into our target compounds, namely, **2Azu**-**5Azu**. Given the high reactivity of 2-bromopentacene, we optimized the synthesis pathway, performing the Sonogashira coupling reaction with 2-bromopentacene-6,13-dione (**1e**) using piperidine as a solvent, achieving **2e** with an impressive yield of 83%. Following a palladium-catalyzed reaction as previously described, coupled with NaBH_4_ reduction and SnCl_2_/AcOH dehydration, we ultimately synthesized **6Azu** successfully with remarkable stability. Bracingly, the implementation of the microwave reaction could significantly reduce the reaction time from 2 days to 4 h to obtain **2Azu**-**5Azu** and **6Azu-pr**. Compounds **2Azu**-**6Azu** all showed good solubility in commonly used organic solvents, such as chloroform, THF, and toluene. Specifically, **2Azu** and **3Azu** were observed to be liquids at room temperature whereas **4Azu**, **5Azu**, and **6Azu** existed in solid form. Thermal gravimetric analysis (TGA, see Supporting Information Figure S2) demonstrated that the 5% weight loss temperatures (*T*_*d*_) of **4Azu**, **5Azu**, and **6Azu** were 250, 341, and 332 °C, respectively, thereby indicative of good thermal stability.

### Quantum Chemical Calculations

Figure 1c displays the three main canonical forms contributing to the stabilization of the singlet ground electronic state of acenes, the covalent and the zwitterionic forms, that overall constitute the closed-shell structure and the diradical forms. Whereas for small and large oligoacenes the covalent closed-shell and diradical forms are respectively dominant, the situation of medium-sized acenes must be described by an amalgam of the three. In this regard, as described above, for the zwitterionic forms of the 6-7-5 **nAzu** isomers, the positive charge of the dipolar structure of the azulene is preferentially delocalized over the acene framework.

**Figure 3:**
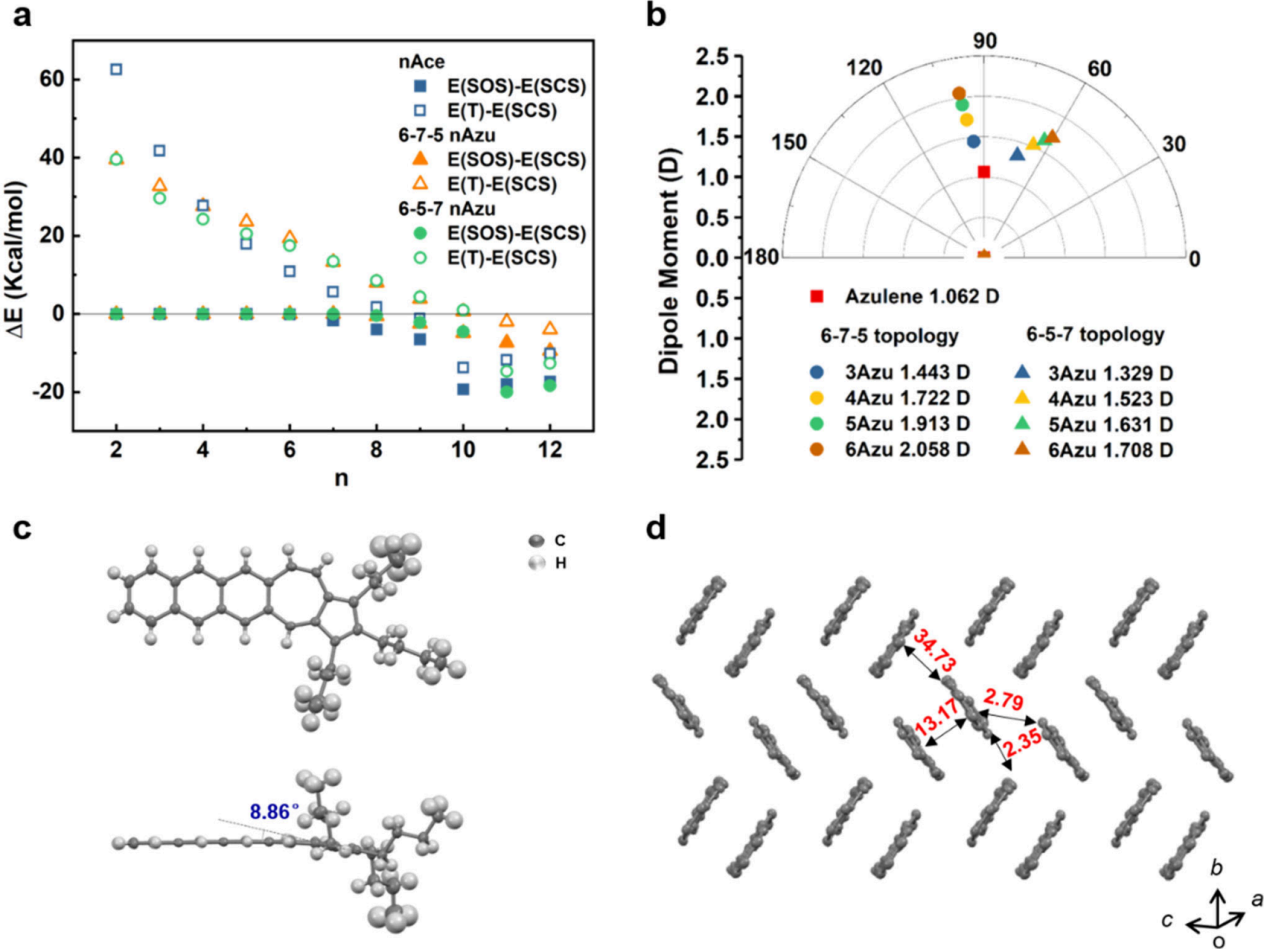
(a) Size-dependent variation of the energy differences between the closed-shell and open-shell forms from restricted and unrestricted broken-symmetry calculations at the (U)B3LYP/6-31G** level of theory as well as the singlet triplet energy differences. SOS, SCS, and T denote the singlet open-shell, singlet closed-shell, and triplet, respectively. (b) Dipolar momentum vectors of **nAzu**s with 6-7-5 or 6-5-7 topology. (c) Molecular structure and dihedral angle of the conjugated core of **5Azu** from singlet crystal diffraction data. (d) Crystal packings from X-ray analysis with calculated transfer integrals of holes of **5Azu**. The red numbers indicate the values of transfer integrals of holes (meV) between adjacent **5Azu** molecules. The alkyl chains are omitted for the sake of clarity.

Quantum chemical calculations have been carried out for 6-7-5 **nAzu** in comparison to those of acenes. For this purpose, the closed-shell to open-shell instability as well as the singlet–triplet energy gaps have been obtained at the DFT/(U)B3LYP/6-31G** level and are disclosed in Figure 3a. We remark that the theoretically evaluated closed-shell structure gives an account of the sum of the closed-shell and zwitterionic forms, whereas the open-shell structure is mostly contributed by the diradical form (for molecules with large diradical character). We observe that the closed-shell structure of acenes is configurationally unstable from hexacene and longer and gives rise to an open-shell diradical ground electronic state which is further characterized by a rather small singlet–triplet gap for longer acenes.

**nAzu** compounds display a similar chain-dependent behavior, but the closed-shell to diradical transition is found for longer molecules, for **7/8Azu** in comparison with the acenes where this point is achieved for pentacene/hexacene. Given that the chemical instability of acenes is proportional to the level of diradical character in their ground electronic state, we can infer that the better stability of 6-7-5 **nAzu** relative to acenes is related to the decrease in the diradical character content. This smaller open-shell character of 6-7-5 **nAzu**s could be due to the stabilization of the zwitterionic form (Figure 1c), in particular, to the favorable delocalization of the positive charge over the electron-rich acene fragment. In Figure 3a, the evolution of the singlet–triplet energy gap is shown for the 6-5-7 **nAzu** isomers, which disclose smaller gaps compared to the current 6-7-5 parents of the same size. This suggests that our 6-7-5 **nAzu** isomers might have improved chemical stability compared to that of the 6-5-7 **nAzu** because of the smaller diradical character.

The UV/vis–NIR absorption spectra of **4Azu** and tetracene together with those of **5Azu** and pentacene in a 10^–5^ M solution in chloroform were monitored over time while being exposed to air under a solar simulator at room temperature or held at 50 °C in the dark. When held at 50 °C in the dark for 12 h, all compounds except **5Azu** showed good thermal stability in chloroform (Figure S3). Besides, as shown in Figure S4, the overall absorbance of tetracene and pentacene rapidly decreased with half-lives of 9 and 0.3 h, respectively. In contrast, **4Azu** and **5Azu** slightly decay with much longer half-lives of 3.5 days and 1 day, respectively, under a solar simulator at room temperature, consistent with the aforementioned evaluation results.

Further extending this reasoning, we can see that the presence of the azulene units in the **nAzu** compounds imparts a unique property which is the appearance of a nonvanishing dipolar momentum in their ground electronic state as a result of the zwitterionic character. The amount of dipolar momentum has been calculated for all compounds in Figure 3b, indicating a progressive increase in the values on increasing the molecular size or number of rings due to the favorable delocalization of the positive charge of the tropylium cation over the acene structure. The enlargement of the separation of the negative and positive charges in longer **nAzu** compounds provokes an enlargement of the dipolar momentum with a maximum value for **6Azu**. This nicely explains that the dipole moment of our series is larger than that of 6-5-7 **nAzu**.

Single crystals suitable for X-ray diffraction analysis were obtained by the slow evaporation method from *n*-hexane, hence the solid-state structure of **5Azu** has been resolved and is represented in Figure 3c,d. We observe the formation of dimers of **5Azu** in the solid which are disposed in perfect face-to-face coupling with the molecules oriented in an antiparallel fashion, which is the way of packing that largely minimizes the electrostatic energy by the antiparallel coupling of the two dipolar momentum vectors in the dimer. This orientation of the **5Azu** molecules further confirms the presence of a more stable zwitterionic form for **5Azu**, as discussed in the previous paragraphs.

### Optical and Electrochemical Properties

**Figure 4:**
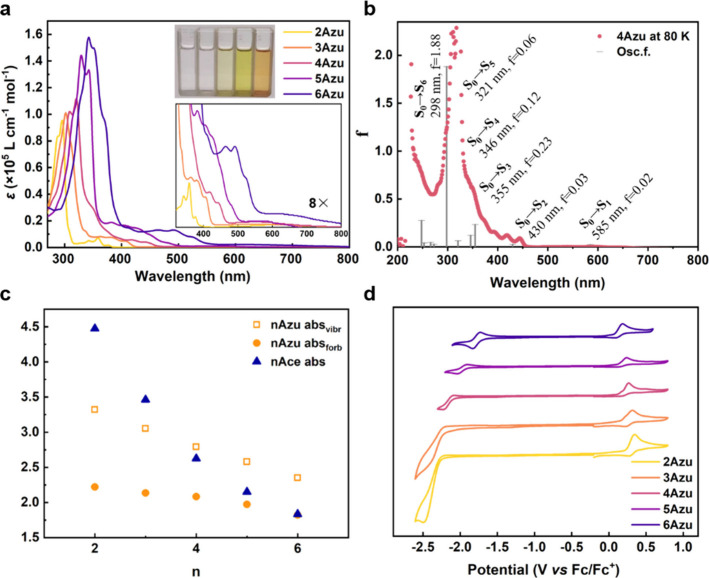
(a) Electronic absorption spectra of **nAzu**s at 298 K in dilute CH_2_Cl_2_ (1.0 × 10^–5^ M). (b) Electronic absorption spectra of **4Azu** at 80 K in 2-methyl THF with simulated TD-DFT B3LYP/6-31G** calculation results. (c) Chain length variation of the energy of the band maxima assigned to the S_0_ → S_1_ (filled circle) and S_0_ → S_2_ (empty square) of **nAzu**s and the S_0_ → S_1_ (filled triangle) of acenes. (d) Cyclic voltammetries of **nAzu**s in dry CH_2_Cl_2_ (1.0 × 10^–3^ M) containing 0.1 M Bu_4_NPF_6_.

Incorporating azulene into acenes leads to a significant perturbation of the molecular electronic structures, resulting in the emergence of dark-yellow colors in **nAzu**s (n = 4–6), which is a notable variation from the corresponding chromatic properties of acenes. Electronic absorption spectra of the compounds are shown in Figure 4a, and Table S2 summarizes the main photophysical and electrochemical data of **nAzu**s.

**4Azu** in Figure 4b displays the highest intensity band at 322 nm with two shoulders. According to the TD-DFT calculations, this band is assigned to an acenic band reproduced by calculations at 298 nm with the largest calculated oscillator strengths (i.e., *f* = 1.88) due to the S_0_ → S_6_ transition. The next experimental absorption bands are a set of three peaks in the range of 350–390 nm which emerge from three different excitations predicted at 321 (*f* = 0.06), 346 (*f* = 0.12) and 355 nm (*f* = 0.23) owing respectively to the S_0_ → S_5_, S_0_ → S_4_, and S_0_ → S_3_ excitations. Between 390 and 440 nm there are three vibronically spaced bands that are associated with the theoretical excitation predicted at 430 nm (*f* = 0.03) and assigned to the S_0_ → S_2_ excitation. Finally, there is a broad absorption band in the visible region peaking at 570 nm and extending up to 650 nm, which is theoretically calculated at 585 nm (*f* = 0.02) owing to the lowest-energy S_0_ → S_1_ transition. This S_0_ → S_1_ band has a common appearance in all compounds and is clearly associated and correlated with the visible absorption band of azulene (i.e., **2Azu**), which corresponds to a charge transfer band, appearing as broad and structureless and originating from the dominant zwitterionic contribution of **2Azu** in its ground electronic state. The electronic absorption spectra of the five compounds share a similar absorption spectral pattern with a progressive red shift of all bands on **2Azu** → **6Azu** which addresses the increased delocalization of the positive charge of the tropylium cation and of the electron density over the acene core, thus contributing to narrowing the optical gap.

The S_0_ → S_2_ band of **nAzu** can be also related to the S_0_ → S_1_ band of acenes (see Supporting Information Figure S17 for the natural transition orbitals), both being weak bands with clear vibronic structure. The maxima wavelength bands of the **nAzu** family display a smaller size dependence variation compared to those of acenes in Figure 4c. Consequently, acenes always display their S_0_ → S_1_ band positioned at wavelengths longer than the S_0_ → S_2_ band of azuacenes with the same number of rings. Comparing these two excitations, the larger optical band gap in long azuacenes can be explained by the larger aromatic character of the 5/7 member rings (i.e., in its zwitterion forms) regarding the comparable benzene rings in acene. From a topological standpoint, the formation of the 7-membered ring brings about an additional fraction of arm-chair structure on the molecular edges, whereas for acenes these are in full zigzag mode, which can topologically address the aperture of the gap (S_0_ → S_2_) in **nAzu**s compared to that in acenes (S_0_ → S_1_).

The cyclic voltammetries of the studied molecules are shown in Figure 4d, where an irreversible oxidation process is detected in **3Azu** that shifts progressively to smaller anodic voltages on increasing size, becoming partially reversible in **6Azu**. Starting with **4Azu**, one cathodic process appears that becomes quasi-reversible in **6Azu** at the same time that a higher reduction potential process emerges on **4Azu** → **6Azu**. **6Azu** can be catalogued as an amphoteric redox molecule, which is a neat distinctive feature compared to acenes. The easy oxidation of **5Azu** will be exploited in the preparation of OFETs in the last section. Accordingly, the highest occupied molecular orbital (HOMO) energy levels increased from −5.04 eV (**2Azu**) to −4.86 eV (**6Azu**), whereas the lowest unoccupied molecular orbital (LUMO) energy levels decreased from −2.51 eV (**2Azu**) to −3.11 eV (**6Azu**). As a result, the calculated HOMO–LUMO energy gap (*E*_g_^CV^) of **2Azu**-**6Azu** is reduced from 2.53 to 1.75 eV.

### Photophysics of **nAzu**s: Steady-State Fluorescence and Picosecond Transient Absorption Spectroscopy, ps-TAS

**Figure 5:**
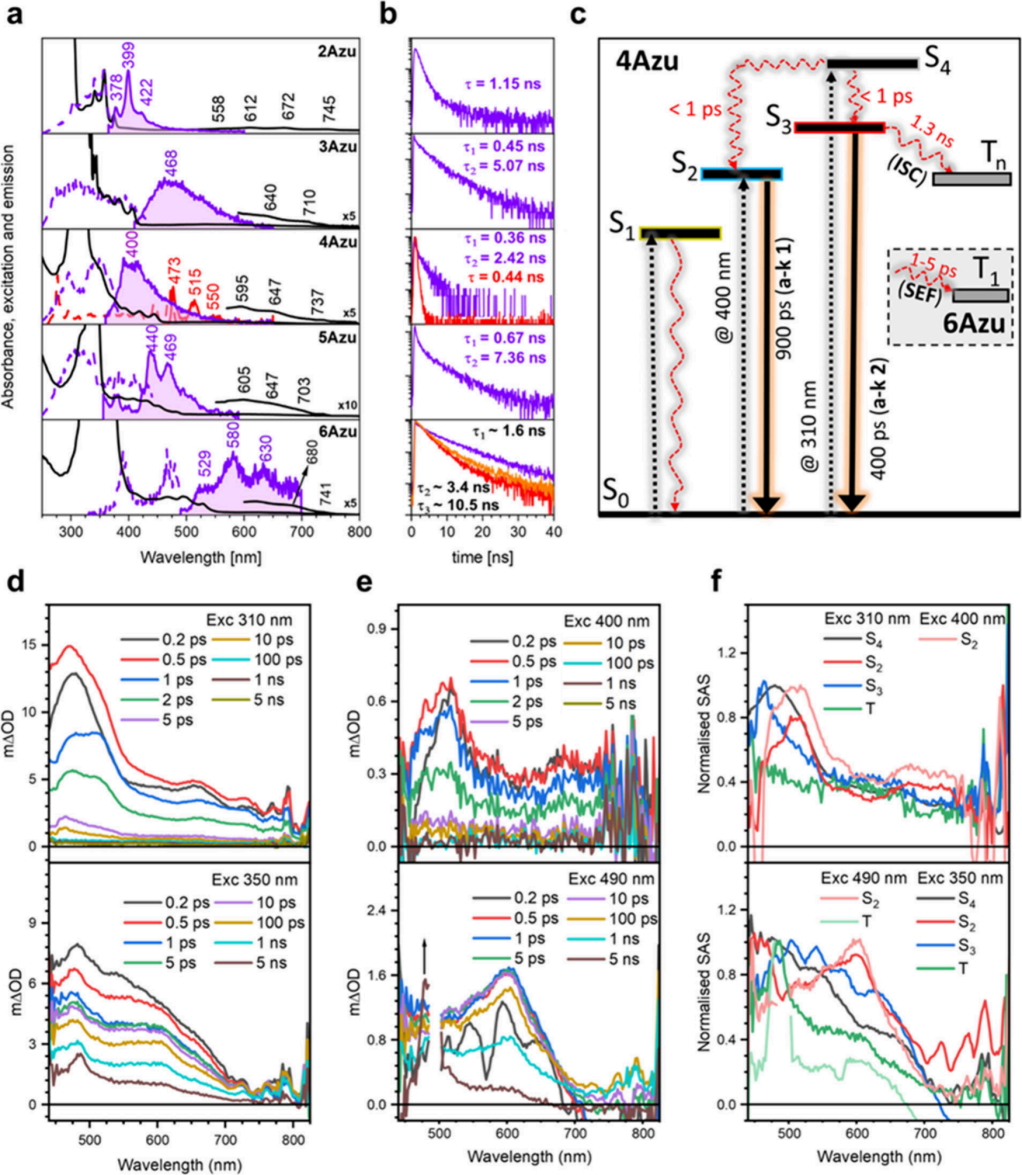
(a) Absorption (black lines), emission (purple filled line), and excitation (purple dashed line) spectra of **2Azu**, **3Azu**, **4Azu**, **5Azu**, and **6Azu** from the top to the bottom all in 10^–5^ M solute concentrations in 2-methyl THF at 80 K. (b) Emission lifetimes for **2Azu**, **3Azu**, **4Azu**, **5Azu**, and **6Azu**. (c) Jablonski diagram of **4Azu** (the inset is the distinctive feature in **6Azu** which produces a triplet by SEF). The position of the energy levels has been calculated by TD-DFT at the B3LYP/6-31G** level of theory. (d, e) Ps-TAS of **4Azu** (top) and **6Azu** (bottom) upon excitation at (d) 310–350 and (e) 400–490 nm. (f) Spectra associated species by global analysis of the ps-TAS spectra for **4Azu** (top) and **6Azu** (bottom). The excitation power was maintained at 0.25 mW.

Figure 5 shows the absorption, emission, and excitation spectra for **nAzu**s at 80 K (see Supporting Information Figures S7 and S8 for the evolution of these spectra with temperature). The fluorescence emission of azulene is one of the textbook examples of the violation of the Kasha rule (i.e., anti-Kasha emission), which consists of the observation of emission features at higher energies (S_2_ → S_0_) than the emission from the first excited S_1_ state (S_1_ → S_0_). The discovery of molecules with anti-Kasha luminescence is now being intensively exploited as a way to manipulate light emission in ampler spectral ranges beyond the constraint of the Kasha rule (i.e., emissions exist only at longer wavelengths than the longest wavelength absorption).^14^ The existence of a large S_2_–S_1_ gap in azulene and the larger oscillator strength of the S_2_ relative to S_0_ are the main ingredients to have anti-Kasha fluorescence in azulene or in our **2Azu** (alkyl-substituted azulene), such as shown in Figure 5, where the anti-Kasha emission (τ = 1.15 ns) of **2Azu** has a great similitude with that of azulene. In **3Azu**, such as in **2Azu**, a neat anti-Kasha emission is also observed which, however, contains two different decays and lifetimes, τ_1_ = 0.45 ns and τ_2_ = 5.07 ns, suggesting that multiple emission are likely to occur. This is confirmed in **4Azu**, where two anti-Kasha emission profiles are detected: (i) by exciting at 340 nm (such as in **2Azu** and **3Azu**), an emission peaking at 400 nm with a long tail of up to 500 nm is recorded with lifetimes of τ_1_ = 0.36 ns and τ_2_ = 2.42 ns. By excitation at wavelengths of up to 450 nm, very similar fractions of emission of the former are detected with the same lifetime. However, (ii) by excitation at 440 nm, a distinctive very well resolved band with several vibronic components with an overall maximum at 473 nm is recorded with a lifetime of τ = 0.44 ns. Hence, **4Azu** is the first member of the azuacene series which clearly documents two anti-Kasha emissions given the lowest energy absorption of **4Azu** at 550–600 nm. Whereas the first emission band at 350 nm is similar and hence related to that of azulene, the second emission band peaking at 473 nm can be termed as tetracene-like anti-Kasha emission since it strongly resembles that of tetracene. This is interesting since the delocalization of the positive charge of the tropylium cation over the acene unit produces a larger dipole moment that might originate from the appearance of a second anti-Kasha emission of tetracene-like form. These features prompt us to define **4Azu** as a sort of superazulene.

The emissions of **5Azu** and **6Azu** are characterized by only one anti-Kasha spectrum that reminds us of the tetracene-like anti-Kasha emission of **4Azu** given the existence of vibronic structure in both, suggesting that these are the main operative excited states (i.e., superazulenic state) involving the acene part such as in **4Azu**. At these emissions, different lifetimes on each compound are detected: (i) for **5Azu**, τ_1_ = 0.67 ns and τ_2_ = 7.36 ns, revealing the involvement of complicated photophysics and the likely presence of two anti-Kasha states which should be converging in just one emission profile and (ii) for **6Azu**, the vibronically resolved emission profile has its main bands at 529/580 nm red-shifted regarding that of **5Azu** (440 and 469 nm), but an increasing degree of complication appears since three different values of lifetimes are measured: τ_1_ = 1.6 ns, τ_2_ = 3.1–3.4 ns, and τ_3_ = 10.0–11.0 ns.

Time-dependent DFT calculations (partially discussed above) are good indicators of the distribution of excited states that might be responsible for the existence of the singular photophysics of **nAzu**s, such as shown in Figure 5 and Supporting Information Figure S15, which display the energies of the low-energy excited states involved in the relevant absorptions/emissions of the acenes and azuacenes. Comparing **2Azu** and naphthalene, the emergence of anti-Kasha emission is related to the appearance of large energy gaps among the three first singlet excited states, which indicates that upon excitation on the S_2_ state, internal conversion is limited by the large S_2_–S_1_ gap increasing the probability of S_2_ → S_0_ light emission. Conversely, in naphthalene, the vanishing S_2_–S_1_ gap produces efficient internal conversion to the S_1_ from which emission occurs (i.e., this explanation is the accepted origin of anti-Kasha emission in azulene). Passing to **4Azu**, as a representative case of the azuacene family, and tetracene, a similar situation is found in comparison with **2Azu**/naphthalene. However, the **4Azu** oscillator strengths indicate a large radiative coupling for the S_2_ and S_3_ states with the ground electronic state, i.e., S_0_ → S_2_ and S_0_ → S_3_ transitions, in comparison to **2Azu**. This large oscillator strength might justify that the two S_2_ and S_3_ states might be uncoupled by internal conversion due to the significant gaps and, at the same time, that each can relax to the ground state by light emission due to the enhanced oscillator strengths.

One interesting point in the emission characteristics of **6Azu** discussed above is related to the long lifetime of one of its emission components. The emission spectrum of **6Azu** must be highlighted in the context of the absence of the fluorescence properties of long acenes. Pentacene and longer acenes do not display emission features due to the activation of highly efficient nonradiative channels associated with the small optical gaps as well as with the participation of diradicaloid states highly promoting fluorescence quenching. Therefore, the hybridization of acenes and azulene in the 6-7-5 topology produces the conservation of photoluminescence in molecules with an increasing number of fused rings (i.e., 6 in **6Azu**) due to the emergence of anti-Kasha properties.

Picosecond transient absorption was carried out to further investigate the excited-state dynamics of these molecules, as shown in Figure 5 and Figures S9–S11. We selected two different pumping excitation wavelengths, *ca*. 300 and 400 nm, to gain insight into the different excited states giving rise to the anti-Kasha emissions discussed above. Herein, we focus on **4Azu**, although similar analysis can be done for all molecules. Excitation of **4Azu** at 310 nm gives rise to an excited-state absorption spectrum (i.e., ESA) characterized by a multiband signal with several maxima at 470, 510, and 660 nm, where each one decays on different time scales. On the other hand, exciting **4Azu** at 400 nm produces a single-band ESA spectrum. Global analysis has been performed to elucidate the different species involved in the photoexcited dynamics. Four different species upon photoexcitation at 310 nm are found, while only one is detected when pumped at 400 nm. Based on the previous band assignment from TD-DFT, excitation at 400 nm populates the S_2_ state from which the S_0_ ground state is recovered. However, excitation at 310 nm populates the S_4_ or S_5_ states, which gives rise to a wide-band ESA spectrum centered at 470 nm with a tail that extends up to 800 nm. This state decays to form two other different ESA spectra featured by a band located at 510 nm, which resembles the ESA spectrum of the S_2_ state (i.e., formed by excitation at 410 nm) and another state with a band at 460 nm which emerges from a larger energy state than the S_2_ state, namely, S_3_. Interestingly, the decay of these two bands is 0.4 and 0.9 ns for S_2_ and S_3_ states, respectively, which are on time scales similar to the lifetimes obtained in the fluorescence experiments. Regarding the fourth species extracted from the global analysis, it displays a featureless ESA profile not revealed in the ps-TAS experiment and that can be assumed to be a long-lived state that can be tentatively assigned to a triplet state formed by intersystem crossing from the S_3_ state. A Jablonski diagram for **4Azu** representing its photoexcited dynamics is depicted in Figure 5c.

For **6Azu**, the ps-TAS experiment upon excitation at 490 nm produced a single-band ESA spectrum centered at 600 nm which strongly resembles the ESA spectrum and peak position of the S_1_ state of tetracene reported at 650 nm. This ESA band of **6Azu** at 600 nm decays on the nanosecond time scale to give rise to a single band component at 475 nm which remains constant during the whole upper time limit of the experiment. This long-lived ESA spectrum might belong to a triplet excited state formed by intersystem crossing. Interestingly, upon excitation of **6Azu** at 350 nm an ESA band at 475 nm appears after a few picoseconds that displays a great similarity with the ESA band of the above-mentioned triplet state, thus suggesting that this triplet state is formed through a very rapid channel when excited at 350 nm compared to the way it emerges when excited at 490 nm. We hypothesize that triplets formed upon 350 nm excitation are created by singlet exciton fission (i.e., SEF). The likely existence of the SEF in **6Azu** is another property of azuacenes inherited from acenes. More work is now underway in our laboratories to demonstrate SEF in these hybrid molecules.

### Organic Field Effect Transistors

**Figure 6:**
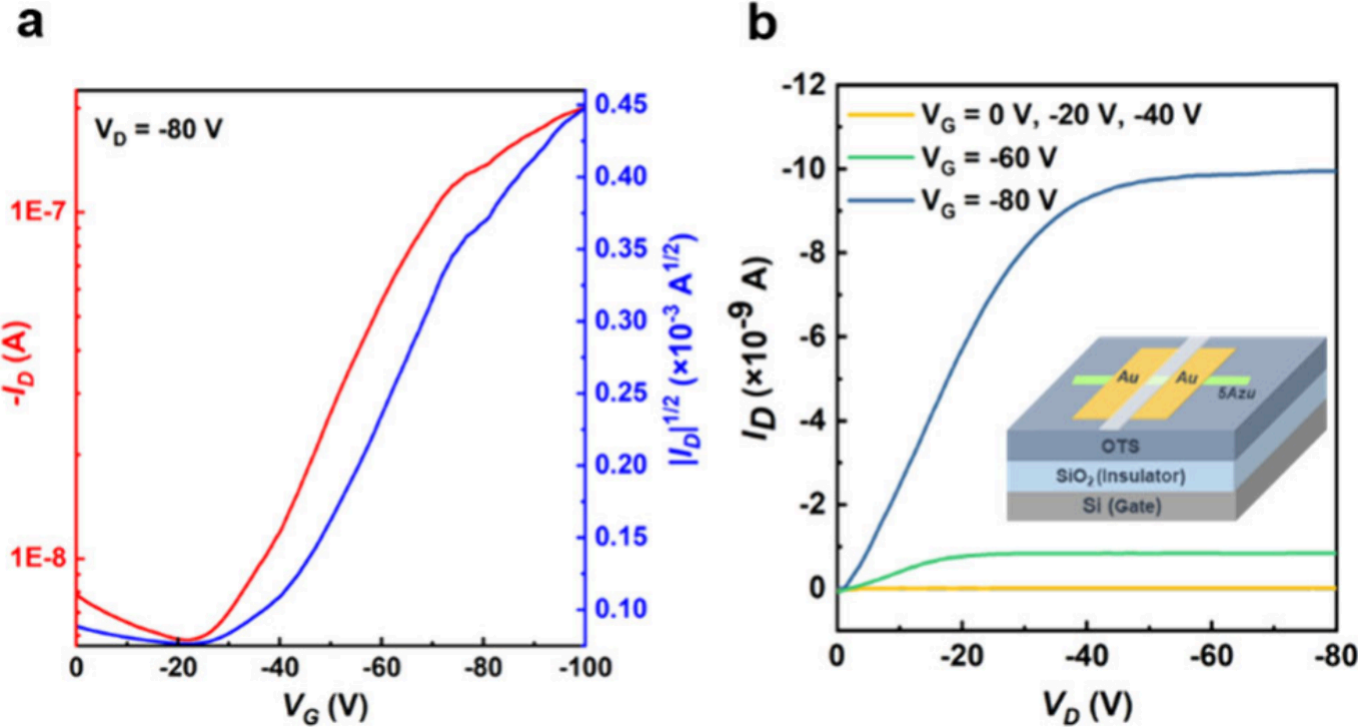
(a) Transport curve and (b) output characteristics based on **5Azu** single-crystal FET devices. Transfer and output characteristics of OFETs were collected in bottom-gate, top-contact single-crystal FET devices using a semiconductor parameter analyzer. Field effect mobility values (μ_sat_) were estimated from the saturation regime using the following equation: −*I*_D_ = (*WC*_i_/2*L*)μ_sat_(*V*_G_ – *V*_th_)^2^. *C*_i_ is the capacitance of the gate insulator, *V*_th_ is the threshold voltage, and *L* and *W* are the length and width of the channel, respectively.

We fabricated single-crystal OFETs using **5Azu** as an example to investigate its charge transport properties. Single crystals of **5Azu** were prepared by drop-casting onto octadecyltrichlorosilane (OTS)-modified SiO_2_ (300 nm)/Si substrates, and then, bottom-gate top-contact (BGTC) OFET devices were prepared using evaporated gold as the source and drain electrodes. The devices based on **5Azu** exhibit typical p-type charge transport characteristics, with the highest hole mobility reaching 0.10 cm^2^ V^–1^ s^–1^, an *I*_*on*_/*I*_*off*_ of 10^5^, and a threshold voltage (*V*_*T*_) of −13.9 V as shown in Figure 6. These results demonstrate that **5Azu** does not improve the electrical performance of pentacene but certainly represents a new family of promising organic semiconducting materials. The hole mobilities of **5Azu** can result from the favorable close contact π–π stacking with large transfer integral values and multiple charge transport routes (Figure 3d). Nonetheless, the relevant feature here of **5Azu** is that it approaches the hole transporting behavior of its parent acene in favor of a description of azuacenes as optimal hybrids of azulene and acenes.

## Conclusions

Azuacene 6-7-5 isomers were conceived from the simple assumption that π-conjugated molecules offer π-paths that are much more suited to stabilize (delocalize) positive charges (than anions), which could be allowed in an isomerization mode of acenes annealed into azulene following the 6-7-5 pattern. In this way, the tropylium cation can easily resonate over the acenes, preserving and further modulating the zwitterionic structure of azulene. The so-designed azuacenes are hybrids of azulene and acenes and have been prepared through a unique reaction protocol that allowed them to be synthesized with a 6-7-5 topology, making the connection of the two 6- and 5-membered moieties by the tropylium ring. This reaction protocol realizes the simultaneous skeletal editing and [3 + 2] annulation to form the azulene moiety. In this manner, **nAzu**s have been synthesized with several lengths of up to 6 rings in a row. We *ca*. categorized **nAzu**s as superazulenes but also as enhanced and diversified acenes given that **nAzu**s take the best properties (i.e., synergy) of azulene and of acenes with the following arguments: (i) for the same number of rings, **nAzu**s are more stable than acenes; (ii) **nAzu**s display the low-energy absorption of azulenes, hence exhibiting enhanced chromic properties compared to those of acenes which are in part modulated by the size of the acene fragment; (iii) from azulene, **nAzu**s present the anti-Kasha emission even with double anti-Kasha bands, which is a unique photonic property given the strict requirements for this to occur; and (iv) **nAzu**s also display the ability to function as hole-transporting semiconductors, by analogy to acenes.

The five synthesized azuacenes balance and optimize the properties of acenes and azulene in a beautiful example of complementarity between fragment molecules and final products which diversify, broaden, and enhance the properties in **nAzu**s compared to those in acenes and azulenes.

## Supporting Information Available

Synthesis details; analytical data obtained by mass spectrometry and NMR; results of single-crystal X-ray analysis, TGA, spectroscopic characterization, transient absorption spectroscopy, theoretical calculations, and OFET device fabrication (PDF)
