# Supplementary material for: Optimal Synergy between Azulenes and Acenes in Azuacenes with 6-7-5 Ring Topology
Source: J Am Chem Soc. 2025 Jan 7;147(2):1574–83. doi: 10.1021/jacs.4c11186 (PMC11744744; doi:10.1021/jacs.4c11186)

Supporting Information

The Optimal Synergy between Azulenes and Acenes in Azuacenes with 6-7-5 Ring Topology

Fei Huang[1,3], Marcos Díaz-Fernández[2,3], José M. Marín-Beloqui[2], Lingyan Sun[1], Yong Chen[1], Shengpei Liu[1], Yuxiang Wang[1], Han Zheng[1], Silu Li[1], Cheng Zhang*[,1], Jingsong You*[,1], and Juan Casado*[,2]

[1]Key Laboratory of Green Chemistry and Technology of Ministry of Education, College of Chemistry, Sichuan University, 29 Wangjiang Road, Chengdu 610064, P. R. China. *e-mail: cheng.zhang@scu.edu.cn; jsyou@scu.edu.cn

[2]Department of Physical Chemistry, University of Malaga, Campus de Teatinos s/n, Málaga, 29071, Spain. *e-mail: casado@uma.es

[3] These authors contributed equally: Fei Huang, Marcos Díaz-Fernández.

Table of Contents

I. General Information S3

II. Synthesis S5

Ⅲ. Single Crystal X-ray Analysis and Crystallographic Data S11

Ⅳ. Stability S13

V. Spectroscopic Characterization S15

VI. Transient Absorption Spectroscopy S18

VII. Theoretical Calculations S21

VIII. OFET Device Fabrication S25

IX. References S26

X. Copies of ^{1}H and ^{13}C NMR Spectra S27

I. General Information

NMR spectra were recorded on an Agilent 400-MR DD2 or Bruker AV11-600MHz spectrometer. The ^{1}H NMR (400/600 MHz) chemical shifts were measured relative to $CDCl_3$ as the internal reference ($CDCl_3$: δ = 7.26 ppm). The ^{13}C NMR (100 MHz or 150 MHz) chemical shifts were given using $CDCl_3$ or Tetrachloroethane-d_2 as the internal standard ($CDCl_3$: δ = 77.16 ppm Tetrachloroethane-d_2: δ = 74.20 ppm;). Gas chromatography mass spectra (GC-MS) were obtained with a Shimadzu GCMS-QP2010SE. Matrix-assisted laser desorption/ionization time of flight mass spectra (MALDI-TOF-MS) were collected with a Shimadzu AXIMA Performance. High-resolution mass spectra (HRMS) were obtained with a Shimadzu LCMS-ITTOF (ESI). X-ray single-crystal diffraction data were collected on an Agilent Technologies Gemini single-crystal diffractometer. Absorption spectra were obtained on a HITACHI U-2910 spectrometer. Cyclic voltammogram (CV) was performed on LK2005A with a solution of tetrabutylammonium hexafluorophosphate (Bu_4NPF_6, 0.1 M) in CH_2Cl_2 as electrolyte and ferrocene/ferrocenium (Fc/Fc^+) as standard. Three-electrode system (Ag/Ag^+, platinum wire and glassy carbon electrode as reference, counter, and work electrode, respectively) was used in the CV measurement. All potentials were corrected against Fc/Fc^+. CV was measured with a scan rate of 100 mV/s. Thermogravimetric analysis (TGA) was carried out using METTLER TOLEDO TGA/DSC2 at a rate of 10 ºC/min under nitrogen atmosphere. Low temperature absorption has been conducted on the Varian Cary 5000 UV-Vis-NIR Spectrophotometer. Emission and excitation spectra were obtained using an Edinburgh Instruments FLS920 Fluorimeter. PL lifetimes were acquired using the Fluorimeter pulsed xenon flash-lamp, Xe900, of 400 mW and Picoquant PLS-λ nanosecond LED diodes. Solutions temperatures were varied from room temperature to 80 K using an Optistat DN Oxford Instrument cryostat. Picosecond transient absorption spectra were obtained using a Helios system from Ultrafast Systems. This setup includes an amplified femtosecond Spectra-Physics Solstice-100F laser, featuring a 128 fs pulse width and a 1 kHz repetition rate, coupled with a Spectra-Physics TOPAS Prime F optical parametric amplifier, covering a range of 195-22000

nm. Samples were studied in degassed 2-methyl-tetrahydrofuran (2-Me-THF, Sigma-Aldrich/Merck, Anhydrous, ≥99%) *ca.* 10^{-3} M solutions.

All the reactions dealing with air- or moisture-sensitive compounds were carried out in a dry reaction vessel under a positive nitrogen pressure. Unless stated otherwise, starting materials were obtained from commercial suppliers and were used without any further purification.

II. Synthesis

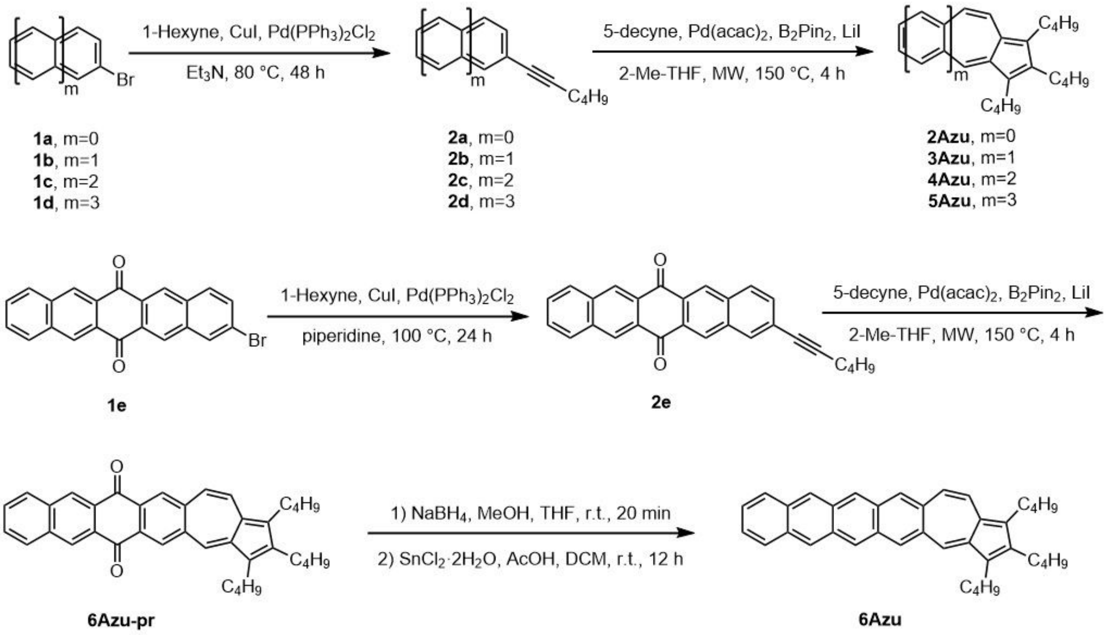

Scheme S1. Synthesis routes for **nAzu**s.

Preparation of the aryl bromides

Bromobenzene (**1a**), 2-bromonaphthalene (**1b**), and 2-bromoanthracene (**1c**) were obtained from commercial suppliers and used without further purification. 2-bromotetracene (**1d**) and 2-bromopentacene-6,13-dione (**1e)** were prepared according to the literature[1,2]. The ^{1}H NMR and ^{13}C NMR data were consistent with the reported data.

Preparation of the aryl alkynes

Hex-1-yn-1-ylbenzene (**2a**) and 2-(hex-1-yn-1-yl) naphthalene (**2b)** were prepared according to the literature.[3]

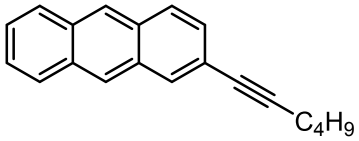

2-(hex-1-yn-1-yl) anthracene (2c)

To a two-neck round-bottom loaded with 2-bromoanthracene (**1c**, 10 mmol, 1.0 equiv) in anhydrous Et_3N under nitrogen atmosphere was added $Pd(PPh_3)_2Cl_2$ (5 mol%), CuI

(10 mol%) and 1-hexyne (1.3 equiv). The resulting solution was stirred at 80 °C for 48 h. The reaction was quenched by addition of aqueous saturated NH_4Cl, the phases were separated and the aqueous phase was extracted with DCM. The combined organics were washed with brine, dried over $MgSO_4$, and concentrated under vacuum. The residue was chromatographed on silica gel with petroleum ether (PE)/DCM = 20/1 (v/v) to provide the corresponding product **2c** as a yellow solid (94% yield). ^{1}H NMR (400 MHz, $CDCl_3$): δ = 8.36 (s, 1H), 8.34 (s, 1H), 8.08 (s, 1H), 8.00-7.97 (m, 2H), 7.91 (d, J = 8.8 Hz, 1H), 7.48-7.45 (m, 2H), 7.41 (d, J = 8.8 Hz, 1H), 2.50 (t, J = 6.8 Hz, 2H), 1.69-1.62 (m, 2H), 1.59-1.49 (m, 2H), 0.99 (t, J = 7.2 Hz, 3H). ^{13}C NMR (100 MHz, $CDCl_3$): δ = 132.1, 132.0, 131.4, 131.3, 130.6, 128.3, 128.3, 128.2, 128.2, 126.2, 126.1, 125.7, 125.7, 121.0, 91.6, 81.3, 31.0, 22.2, 19.4, 13.8. EI-MS: calcd for $C_{20}H_{18}$: $[M]^+$, 258.1, found: 258.1.

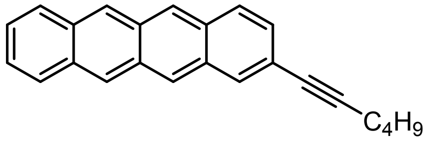

2-(hex-1-yn-1-yl) tetracene (2d)

A two-neck round-bottom with a magnetic stir bar was charged with 2-bromotetracene (**1d**, 8.8 mmol, 1.0 equiv), $Pd(PPh_3)_2Cl_2$ (5 mol%), CuI (10 mol%), degassed Et_3N (88 mL) and 1-hexyne (1.3 equiv). The resulting mixture was stirred at 80 °C for 48 h. After cooling the solid was separated by filtration and washed with water, methanol and CH_2Cl_2 and dried under vacuum. **2d** was obtained as an orange solid (82 % yield). ^{1}H NMR (400 MHz, $CDCl_3$): δ = 8.63 (s, 2H), 8.59 (s, 1H), 8.57 (s, 1H), 8.06 (s, 1 H), 7.99 (dd, J = 6.8, 3.2 Hz, 2H), 7.90 (d, J = 8.8 Hz, 1H), 7.40 (dd, J = 6.8, 3.2 Hz, 2H), 7.33 (d, J = 8.8 Hz, 1H), 2.50 (t, J = 7.20 Hz, 2H), 1.70-1.63 (m, 2H), 1.57-1.50 (m, 2H), 0.99 (t, J = 7.6 Hz, 3H). ^{13}C NMR (100 MHz, $CDCl_3$): δ = 131.8, 131.5, 131.1, 130.6, 130.5, 130.4, 128.4, 128.3, 127.9, 126.6, 126.5, 126.4, 126.2, 125.4, 120.7, 92.0, 81.5, 31.1, 22.3, 19.5, 13.9. MALDI-TOF-MS: calcd for $C_{24}H_{20}$: $[M]^+$, 308.2, found: 308.6.

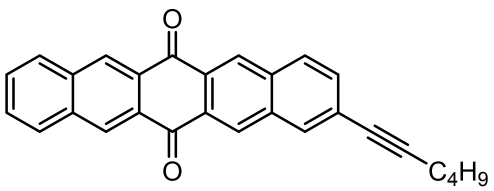

2-(hex-1-yn-1-yl) pentacene-6,13-dione (2e)

A two-neck round-bottom with a magnetic stir bar was charged with 2-bromopentacene-6,13-dione (**1e**, 2 mmol, 1.0 equiv), $Pd(PPh_3)_2Cl_2$ (5 mol%), CuI (10 mol%), degassed piperidine (18 mL) and 1-hexyne (1.3 equiv). The resulting mixture was stirred at 100 ºC for 24 h. After cooling the solid was separated by filtration and washed with water, methanol and CH_2Cl_2 and dried under vacuum. **2e** was obtained as a pale yellow solid in 83 % yield. ^{1}H NMR (400 MHz, $CDCl_3$): δ = 8.91 (s, 2H), 8.85 (s, 1H), 8.82 (s, 1H), 8.12-8.10 (m, 3H), 8.00 (d, J = 8.4 Hz, 1H), 7.70 (dd, J = 6.4, 3.2 Hz, 2H), 7.63 (d, J = 8.8 Hz, 1H), 2.50 (t, J = 7.20 Hz, 2H), 1.70-1.62 (m, 2H), 1.57-1.49 (m, 2H), 0.99 (t, J = 7.2 Hz, 3H). ^{13}C NMR (100 MHz, $CDCl_3$): δ = 183.0, 182.9, 135.4, 135.4, 135.1, 134.2, 132.9, 132.5, 131.2, 130.8, 130.7, 130.6, 130.3, 130.0, 130.0, 129.9, 129.6, 129.6, 129.4, 125.7, 94.3, 80.4, 30.8, 22.2, 19.5, 13.8. MALDI-TOF-MS: calcd for $C_{24}H_{20}$: $[M]^+$, 308.2, found: 308.6.

General procedure for the synthesis of 2Azu-5Azu and 6Azu-pr.

A dried Schlenk tube with a magnetic stir bar was charged with alkyne **2a-2e** (0.2 mmol, 1.0 equiv), $Pd(acac)_2$ (10 mol%), B_2pin_2 (4.0 equiv), LiI (6.0 equiv), 2-Me-THF (2 mL) and 5-decyne (3.0 equiv) under N_2. The resulting solution was stirred at 150 ºC for 4 h under microwave irradiation. The solution was filtered through a Celite pad and washed with DCM. The filtrate was concentrated under vacuum and the residue was purified by column chromatography on silica gel to provide the desired product.

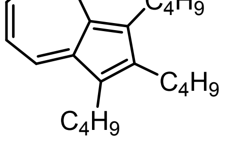

1,2,3-tributylazulene (2Azu)

Purification on a silica-gel column chromatography with PE afforded the desired product **2Azu** as a blue liquid (31% yield). ^{1}H NMR (600 MHz, $CDCl_3$): δ = 8.10 (d, J = 9.6 Hz, 2H), 7.38 (t, J = 9.6 Hz, 1H), 6.95 (t, J = 9.6 Hz, 2H), 3.01 (t, J = 7.8 Hz, 4H), 2.91 (t, J = 7.8 Hz, 2H), 1.63-1.57 (m, 6H), 1.52-1.43 (m, 6H), 1.01-0.96 (m, 9H). ^{13}C NMR (100 MHz, $CDCl_3$): δ = 151.4, 135.8, 135.6, 131.3, 128.3, 120.6, 34.7, 33.6, 27.5, 25.5, 23.6, 23.4, 14.3, 14.2. HRMS (ESI$^+$): calcd for $C_{22}H_{33}^+$: [M+H]$^+$, 297.2577, found: 297.2577.

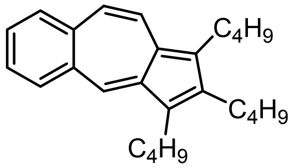

1,2,3-tributylbenzo[*f*]azulene (3Azu)

Purification on a silica-gel column chromatography with PE afforded the desired product **3Azu** as a purple liquid (36% yield). ^{1}H NMR (400 MHz, $CDCl_3$): δ = 8.04 (s, 1H), 7.88 (d, J = 8.0 Hz, 1H), 7.68 (d, J = 8.0 Hz, 1H), 7.56 (d, J = 11.6 Hz, 1H), 7.50 (t, J = 7.6 Hz, 1H), 7.42 (t, J = 8.0 Hz, 1H), 6.98 (d, J = 11.6 Hz, 1H), 2.93 (t, J = 8.0 Hz, 2H), 2.85 (t, J = 8.0 Hz, 2H), 2.75 (t, J = 7.6 Hz, 2H), 1.65-1.58 (m, 4H), 1.54-1.41 (m, 8H), 1.00-0.95 (m, 9H). ^{13}C NMR (100 MHz, $CDCl_3$): δ = 147.3, 140.8, 138.4, 136.2, 135.0, 134.3, 132.3, 131.5, 129.4, 128.8, 126.8, 126.3, 125.8, 123.0, 35.3, 33.6, 33.4, 27.0, 25.6, 25.1, 23.5, 23.3, 23.3, 14.3, 14.2, 14.2. HRMS (ESI$^+$): calcd for $C_{26}H_{35}^+$: [M+H]$^+$, 247.2733, found: 247.2733.

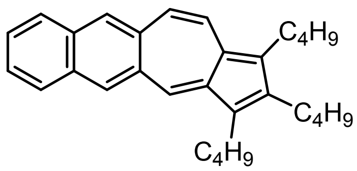

1,2,3-tributylnaphtho[2,3-*f*]azulene (4Azu)

Purification on a silica-gel column chromatography with PE afforded the desired product **4Azu** as a dark green solid (38% yield). ^{1}H NMR (600 MHz, $CDCl_3$): δ = 8.32 (s, 1H), 8.06 (s, 1H), 7.96 (d, J = 8.4 Hz, 1H), 7.94 (s, 1H), 7.91 (d, J = 8.4 Hz, 1H), 7.53 (t, J = 6.6 Hz, 1H), 7.49 (t, J = 7.8 Hz, 1H), 7.27 (d, J = 12.0 Hz, 1H), 6.96 (d, J =

11.4 Hz, 1H), 2.86 (t, J = 8.4 Hz, 2H), 2.76 (t, J = 8.4 Hz, 2H), 2.64 (t, J = 7.8 Hz, 2H), 1.65-1.57 (m, 4H), 1.55-1.43 (m, 8H), 1.02-0.96 (m, 9H). ^{13}C NMR (100 MHz, $CDCl_3$): δ = 145.5, 141.8, 140.3, 135.9, 134.8, 134.1, 133.3, 131.6, 131.6, 130.8, 130.6, 128.0, 127.5, 127.2, 125.9, 125.8, 124.7, 123.5, 35.2, 33.4, 33.2, 26.7, 25.7, 25.0, 23.5, 23.4, 23.4, 14.3, 14.2, 14.2. HRMS (ESI$^+$): calcd for $C_{30}H_{37}^+$: [M+H]$^+$, 397.2890, found: 397.2891.

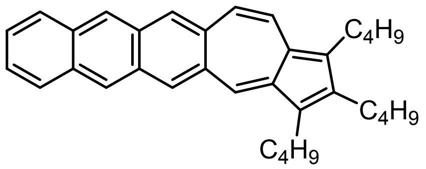

1,2,3-tributylazuleno[5,6-*b*]anthracene (5Azu)

Purification on a silica-gel column chromatography with PE afforded the desired product **5Azu** as a dark green solid (16% yield). ^{1}H NMR (400 MHz, $CDCl_3$): δ = 8.54 (s, 1H), 8.46 (s, 1H), 8.45 (s, 1H), 8.16 (s, 1H), 8.04-8.00 (m, 2H), 7.81 (s, 1H), 7.50-7.44 (m, 2H), 7.11 (d, J = 11.6 Hz, 1H), 6.89 (d, J = 12.0 Hz, 1H), 2.81 (t, J = 7.6 Hz, 2H), 2.71 (t, J = 7.6 Hz, 2H), 2.59 (t, J = 7.6 Hz, 2H), 1.67-1.58 (m, 3H), 1.55-1.42 (m, 9H), 1.04-0.96 (m, 9H). ^{13}C NMR (100 MHz, $CDCl_3$): δ = 144.8, 142.5, 142.1, 135.4, 135.4, 134.0, 132.7, 132.2, 131.8, 131.7, 131.3, 130.4, 130.2, 128.5, 128.3, 126.6, 126.0, 125.9, 125.6, 125.5, 124.0, 123.8, 35.1, 33.3, 33.1, 26.5, 25.7, 25.0, 23.4, 23.4, 14.3, 14.2, 14.2. HRMS (ESI$^+$): calcd for $C_{34}H_{39}^+$: [M+H]$^+$, 447.3046, found: 447.3046.

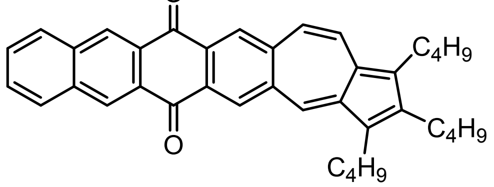

1,2,3-tributylazuleno[5,6-*b*]tetracene-6,13-dione (6Azu-pr)

Purification on a silica-gel column chromatography with PE/DCM = 2/1 (v/v) afforded the desired product **6Azu-pr** as a purple solid (39% yield). ^{1}H NMR (400 MHz, $CDCl_3$): δ = 8.91 (s, 1H), 8.89 (s, 1H), 8.87 (s, 1H), 8.63 (s, 1H), 8.25 (s, 1H), 8.12-8.10 (m, 2H), 7.72 (d, J = 11.2 Hz, 1H), 7.70 (dd, J = 6.0, 3.2 Hz, 2H), 7.21 (d, J = 11.6 Hz, 1H), 2.98 (t, J = 7.6 Hz, 2H), 2.88 (t, J = 8.0 Hz, 2H), 2.77 (t, J = 7.6 Hz, 2H), 1.69-1.43 (m,

12H), 1.04-0.97 (m, 9H). ^{13}C NMR (100 MHz, CDCl$_3$): δ = 182.9, 182.3, 177.0, 150.3, 142.8, 142.0, 140.2, 135.6, 135.4, 135.3, 134.6, 133.5, 132.5, 132.4, 132.2, 130.7, 130.6, 130.3, 130.0, 129.8, 129.7, 129.6, 129.5, 129.3, 127.3, 122.9, 35.2, 33.6, 33.2, 27.1, 25.8, 25.3, 23.5, 23.4, 23.4, 14.3, 14.2, 14.1. HRMS (ESI$^+$): calcd for $C_{38}H_{39}O_2^{+}$: [M+H]$^+$, 527.2945, found: 527.2943.

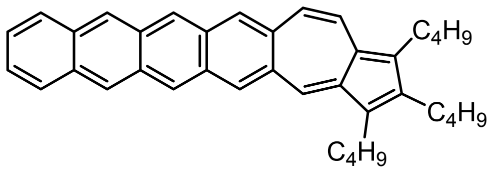

1,2,3-tributylazuleno[5,6-*b*]tetracene (6Azu)

To a 100 mL two-neck round-bottom flask was added **6Azu-pr** (0.1 mmol, 1.0 equiv), MeOH (2 mL) and THF (2 mL). The mixture was cooled to 0 °C and $NaBH_4$ (12.0 equiv) was added. The mixture was stirred at room temperature for 20 min, and then H_2O (2 mL) was added. The organic layer was separated, washed with distilled water and brine, dried over Mg_2SO_4, and concentrated in vacuo. The green solid was suspended in DCM (2 mL) and then AcOH (1 mL) and $SnCl_2{\cdot}2H_2O$ (2.4 equiv) were added. The mixture was stirred at room temperature for 12 h and then saturated $NaHCO_3$ was added. The phases were separated and the aqueous phase was extracted with DCM. The combined organics were washed with water and brine, dried over $MgSO_4$, and concentrated under vacuum. The residue was chromatographed on silica gel with PE/DCM = 10/1 (v/v) to provide the corresponding product **6Azu**. Product was obtained as a dark purple solid (43% yield). ^{1}H NMR (400 MHz, CDCl$_3$): δ = 8.78 (s, 1H), 8.69 (s, 2H), 8.67 (s, 1H), 8.42 (s, 1H), 8.12 (s, 1H), 8.02-7.99 (m, 2H), 7.71 (s, 1H), 7.42-7.40 (m, 2H), 7.01 (d, J = 12.0 Hz, 1H), 6.82 (d, J = 11.6 Hz, 1H), 2.77 (t, J = 7.6 Hz, 2H), 2.67 (t, J = 7.6 Hz, 2H), 2.55 (t, J = 7.2 Hz, 2H), 1.64-1.43 (m, 12H), 1.04-0.96 (m, 9H). ^{13}C NMR (150 MHz, Tetrachloroethane-d_2): 145.0, 144.3, 142.4, 135.9, 134.0, 132.7, 131.7, 131.1, 130.4, 130.4, 130.2, 128.6, 128.6, 127.1, 127.0, 126.6, 125.9, 125.9, 125.7, 125.7, 124.0, 124.0, 120.6, 99.8, 35.2, 33.4, 33.2, 32.2, 30.0, 26.6, 25.9, 23.6, 23.5, 23.5, 23.1, 14.6, 14.5, 14.5. HRMS (ESI$^+$): calcd for $C_{38}H_{41}^{+}$: [M+H]$^+$, 497.3203, found: 497.3201.

Ⅲ. Single Crystal X-ray Analysis and Crystallographic Data

The single crystals of compound **5Azu** obtained from slow solvent evaporation method from n-hexane.

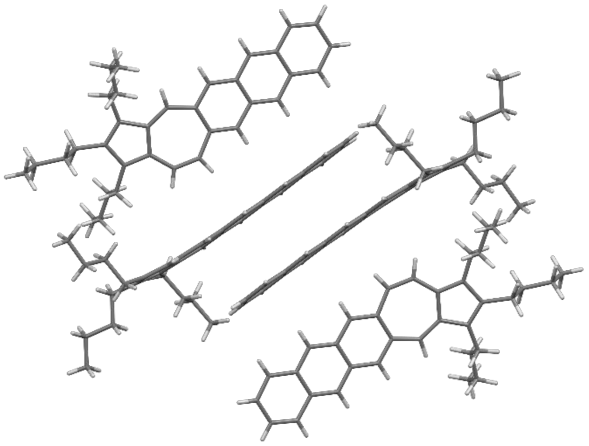

Figure S1. Crystal X-ray structure of **5Azu**.

Table S1. Crystal data and structure refinement for **5Azu**.

Compound	5Azu
Formula	$C_{34}H_{38}$
Molecular Weight	446.64
Temperature [K]	292(2)
Crystal System	Monoclinic
Crystal Symmetry	C_{2h}
Space Group	$P2_1/c$
a [Å]	24.666(5)
b [Å]	11.355(2)
c [Å]	9.3676(17)
α [°]	90
β [°]	99.394(6)
γ [°]	90
V [Å^3]	2588.5(8)
Z	4
Density [g cm^{-3}]	1.146
R [%]	6.86
R_w [%]	15.31
μ [mm^{-1}]	0.064
F [000]	968

Crystal size [mm^3]	0.10 × 0.23 × 0.50
Radiation	MoKα (λ = 0.71073)
2Θ range for data collection [°]	3.958 to 50.000
Index ranges	$-29 \leq h \leq 29$, $-13 \leq k \leq 13$, $-9 \leq l \leq 11$
Reflections collected	35763
Independent reflections	4555 [R_{int} = 0.0686, R_{sigma} = 0.1191]
Data/restraints/parameter	4555/0/310
Goodness-of-fit on F^2	1.032
Final R indexes [I>=2σ (I)]	R_1 = 0.0686, wR_2 = 0.1531
Final R indexes [all data]	R_1 = 0.1191, wR_2 = 0.1865
Largest diff. peak/hole [e $Å^{-3}$]	0.46/-0.31

Ⅳ. Stability

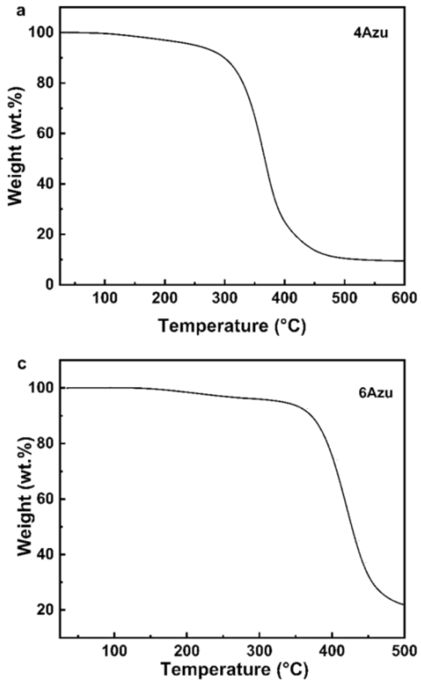

Figure S2. Thermal gravimetric analysis (TGA) curves of compounds (**a**) **4Azu**, (**b**) **5Azu**, and (**c**) **6Azu**.

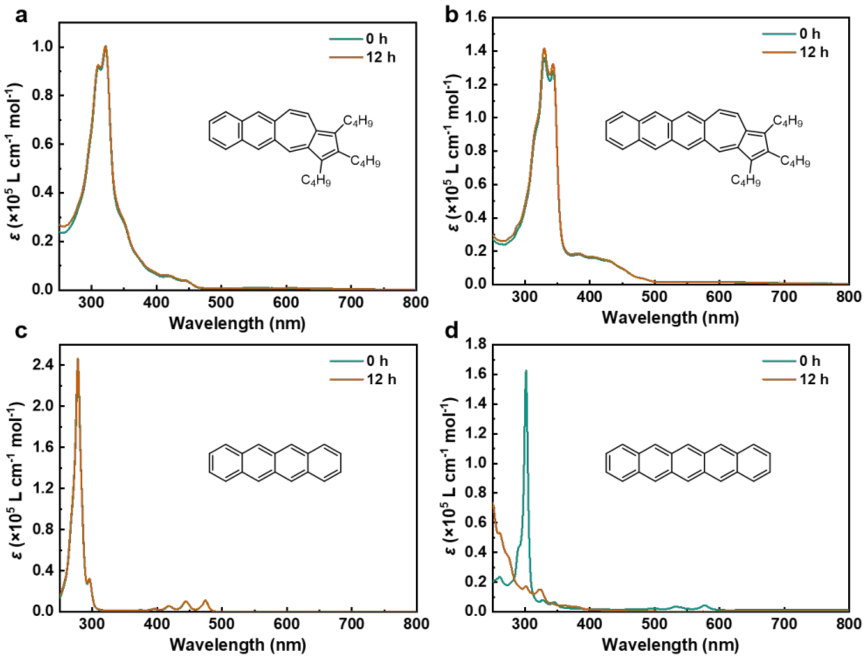

Figure S3. Thermal stability evaluation of (a) **4Azu**, (b) **5Azu**, (c) tetracene, and (d) pentacene. These compounds were dissolved in a 10^{-5} M chloroform solution and heated at 50 °C in the dark for 12 h with ambient air.

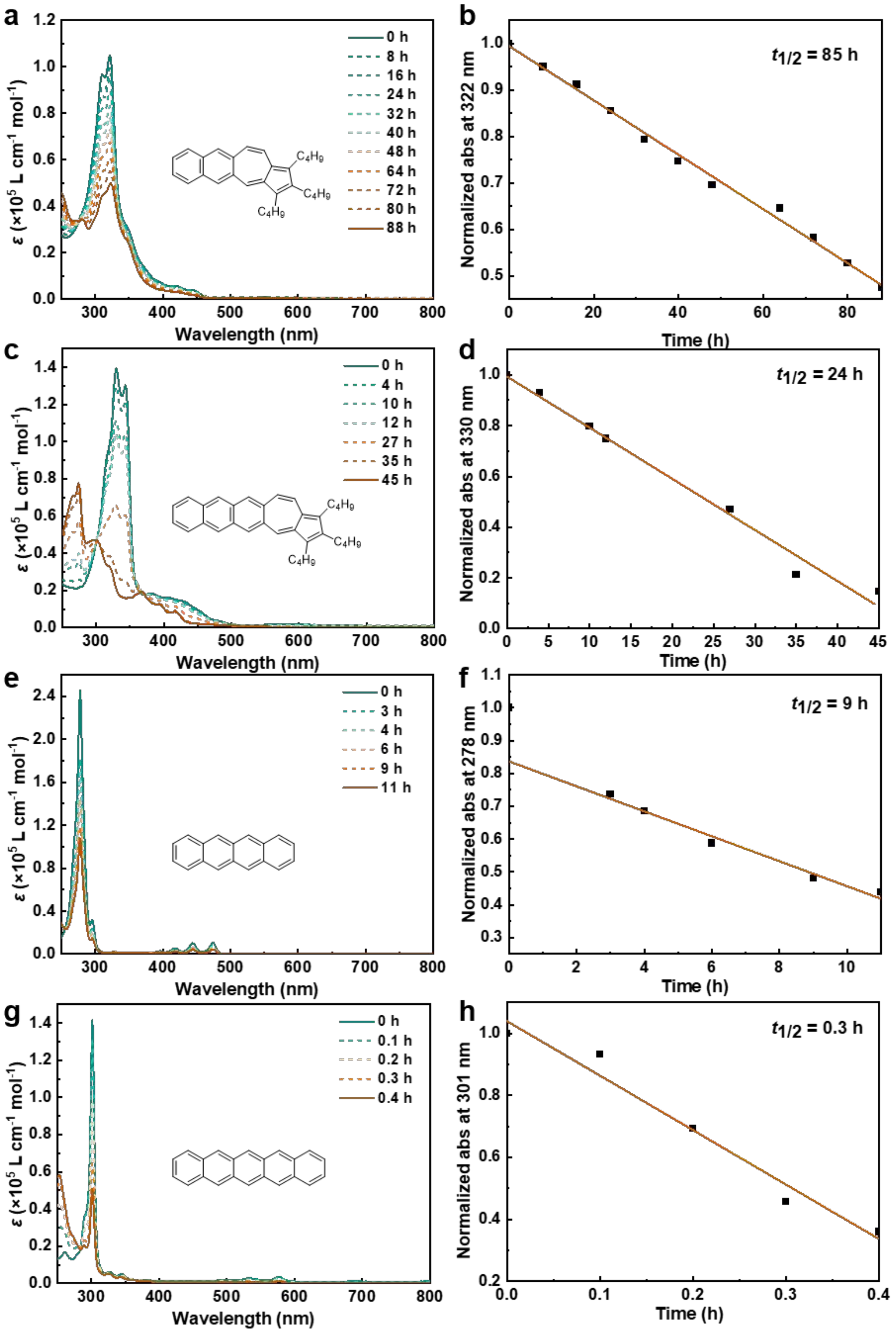

Figure S4. Photo stability evaluation of (a-b) **4Azu**, (c-d) **5Azu**, (e-f) tetracene, and (g-h) pentacene. These compounds were dissolved in a 10^{-5} M chloroform solution under a solar simulator at room temperature with ambient air.

V. Spectroscopic Characterization

Table S2. Photophysical and electrochemical properties of **nAzu**s (n = 2-6) in dilute CH_2Cl_2 solution.

Compounds	λ_{max}[a] (nm)	ε_{max} ($L \cdot mol^{-1} \cdot cm^{-1}$)	HOMO[b] (eV)	LUMO[b] (eV)	$E_g^{CV[c]}$ (eV)
**2Azu**	377	0.955	-5.04	-2.51	2.53
**3Azu**	407	1.012	-4.96	-2.64	2.32
**4Azu**	444	1.118	-4.91	-2.72	2.19
**5Azu**	481	1.444	-4.90	-2.90	2.00
**6Azu**	525	1.578	-4.86	-3.11	1.75

[a] Absorption maxima in CH_2Cl_2 (1.0×10^{-5} M). [b] From CVs measured in CH_2Cl_2 (1.0×10^{-3} M). The HOMO and LUMO energy levels are adjusted according to the redox half potential of Fc/Fc^+ and estimated according to the formula: E_{HOMO} (eV) = $-(4.8 + E^{ox} - E_{(Fc/Fc^+)}{}^{1/2})$, E_{LUMO} (eV) = $-(4.8 + E^{red} - E_{(Fc/Fc^+)}{}^{1/2})$. [c] Estimated according to the formula: E_g (eV) = $E_{LUMO} - E_{HOMO}$.

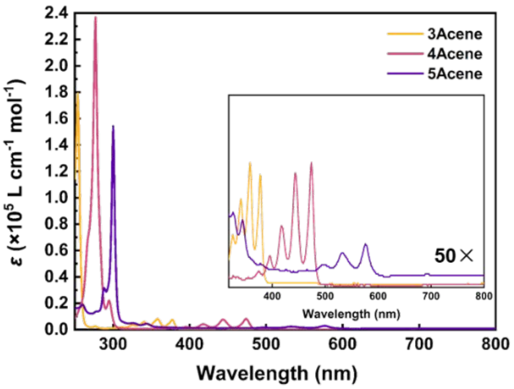

Figure S5. UV-vis absorption spectra of **nAces** (n = 3-5) in dilute CH_2Cl_2 (1.0×10^{-5} M).

Table S3. UV-vis absorption data of **nAces** (n = 3-5) in dilute CH_2Cl_2 (1.0×10^{-5} M).

Compounds	Absorption wavelength (ε)
**3Ace**	254 nm (1.791); 277 nm (0.025); 31 1 nm (0.018); 325 nm (0.035); 341 nm (0.059); 358 nm (0.083); 377 nm (0.075)
**4Ace**	277 nm (2.368); 295 nm (0.221); 374 nm (0.011); 395 nm (0.021); 417 nm (0.041); 443 nm (0.076);474 nm (0.083)
**5Ace**	260 nm (0.191); 288 nm (0.317); 300 nm (1.543); 326 nm (0.050);344 nm (0.045); 495 nm (0.015); 532 nm (0.023); 576 nm (0.029)

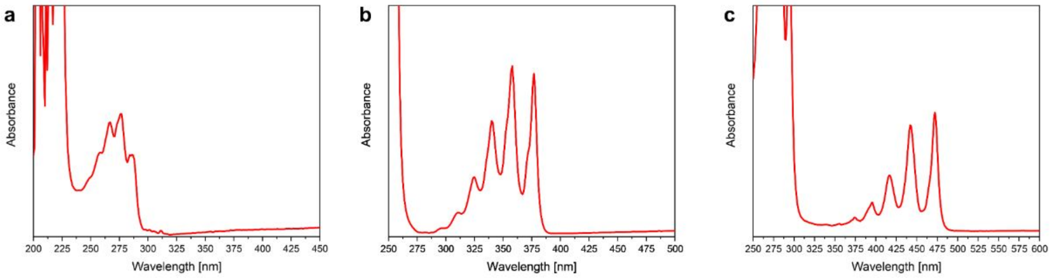

Figure S6. Electronic absorption spectra for **nAcene** (**a** n= 2, **b** n= 3, **c** n=4) in 2-MeTHF.

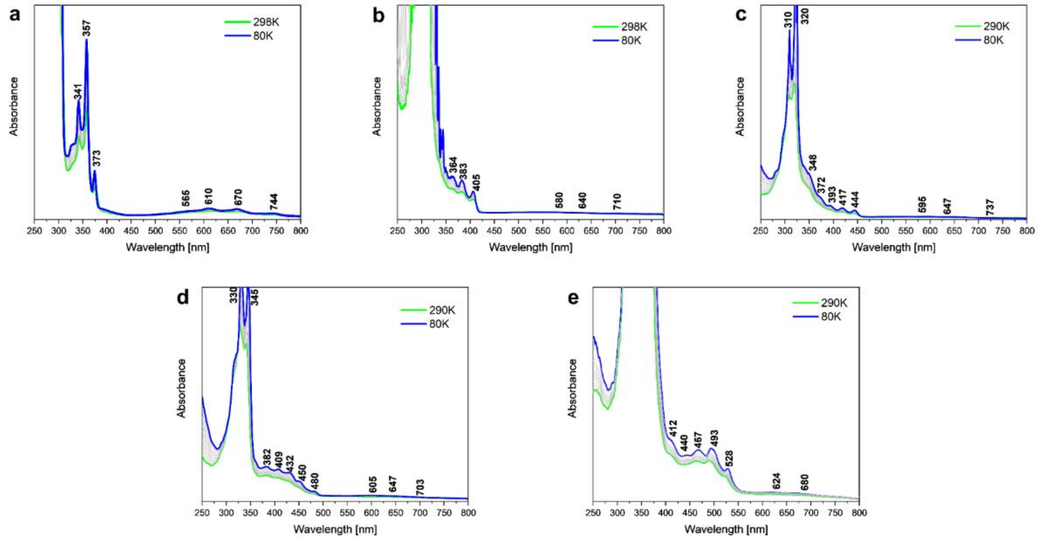

Figure S7. Absorption spectra for **nAzu** (**a** n= 2, **b** n= 3, **c** n=4, **d** n= 5, **e** n=6) in 2-MeTHF obtained from 298 to 80 K.

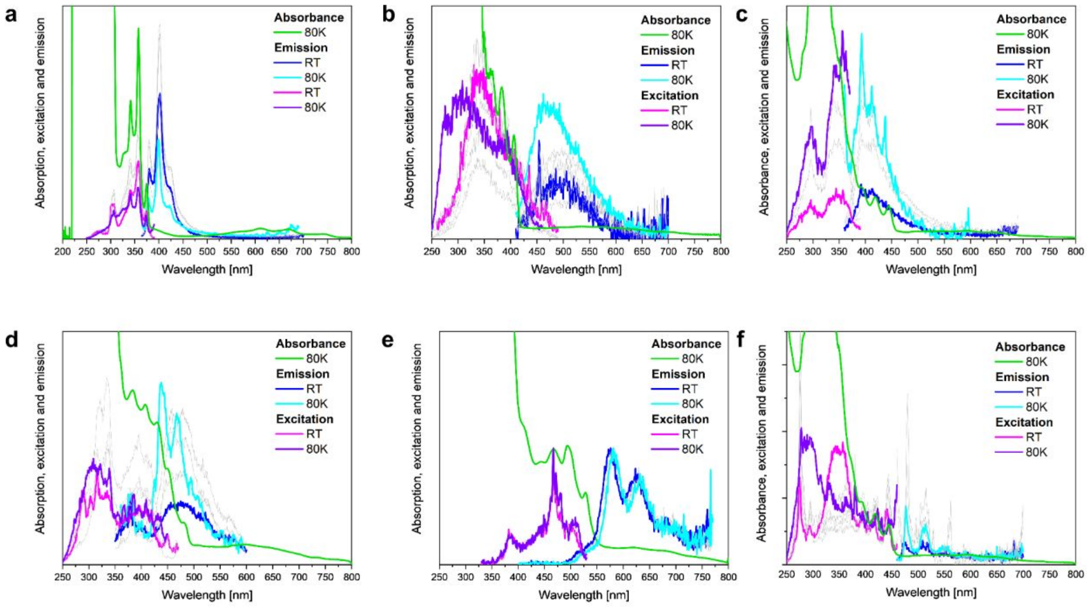

Figure S8. Emission (blue) and excitation (pink) spectra obtained for **nAzu** (**a** n= 2, **b** n= 3, **c** and **f** n=4, **d** n= 5, **e** n=6) in 2-MeTHF from room temperature to 80 K. Absorbance spectra (green) at 80 K has been added for each **nAzu** to compare with the excitation spectra.

Table S4. Fluorescence lifetimes of **nAzu** (n = 2-6).

	**nAzu**	
**n**	**λ_{exc} (nm)**	**$\tau_{1/2}$ (ns)**
**2**	340	1.15
**3**	340	0.45, 5.07
**4**	340<br>450	0.36, 2.42<br>0.44
**5**	340	0.67, 7.36
**6**	475	1.58, 9.52,<br>3.38, 10.6<br>3.17, 11.2

VI. Transient Absorption Spectroscopy

2Azu

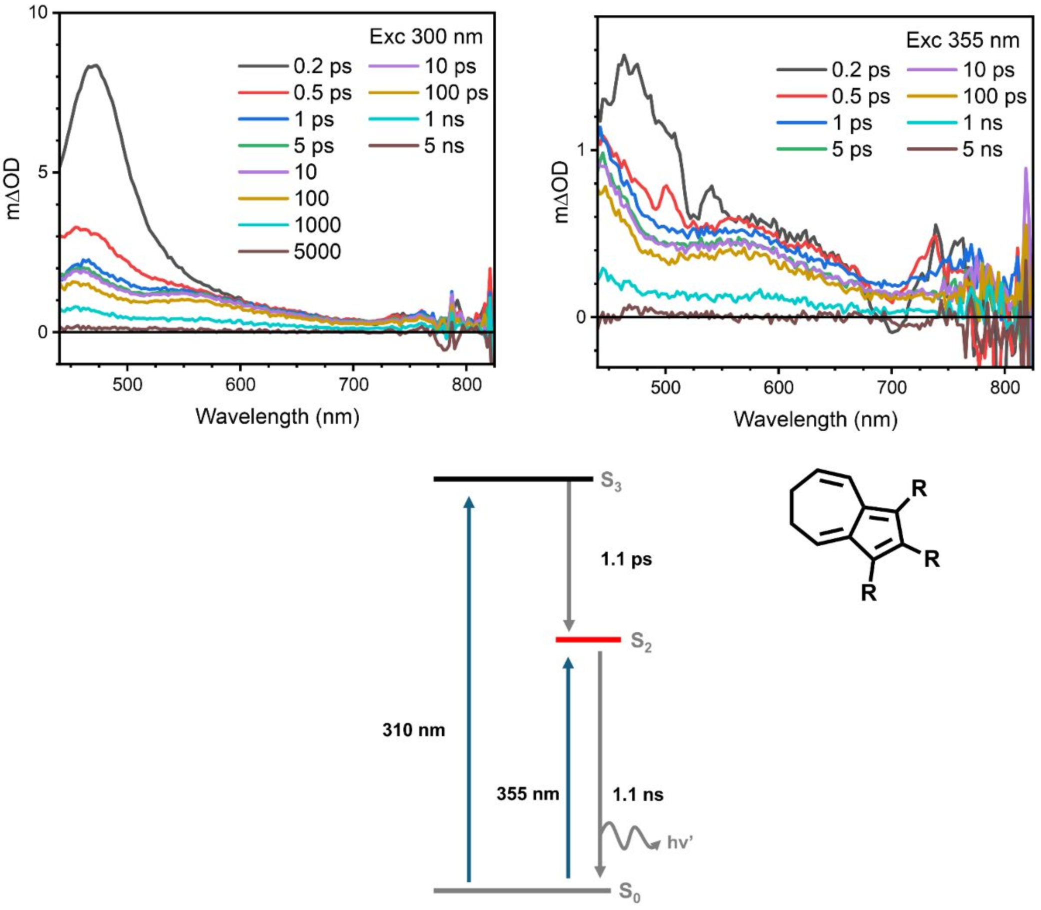

Figure S9. Ps-TAS characterization of **2Azu** upon excitation at (**a**) 300 and (**b**) 355 nm. Excitation power has been maintained at 0.25 mW. (**c**) Jablonski diagram indicating the excited state dynamics of **2Azu** upon excitation.

Excitation of **2Azu** at 355 nm shows a feature with a larger band at 450 nm and a smaller one at 580 nm. This ESA decays in 1.1 ns, matching with the **2Azu** fluorescence lifetime of (1.15 ns). We, therefore, associate this species with the S_2 state, the one showing the fluorescence in pristine azulene. On the other hand, exciting **2Azu** with higher energy, at 310 nm, a larger single band at 470 nm that swiftly disappears in the first picosecond which we, hence, assign to a S_3 state.

3Azu

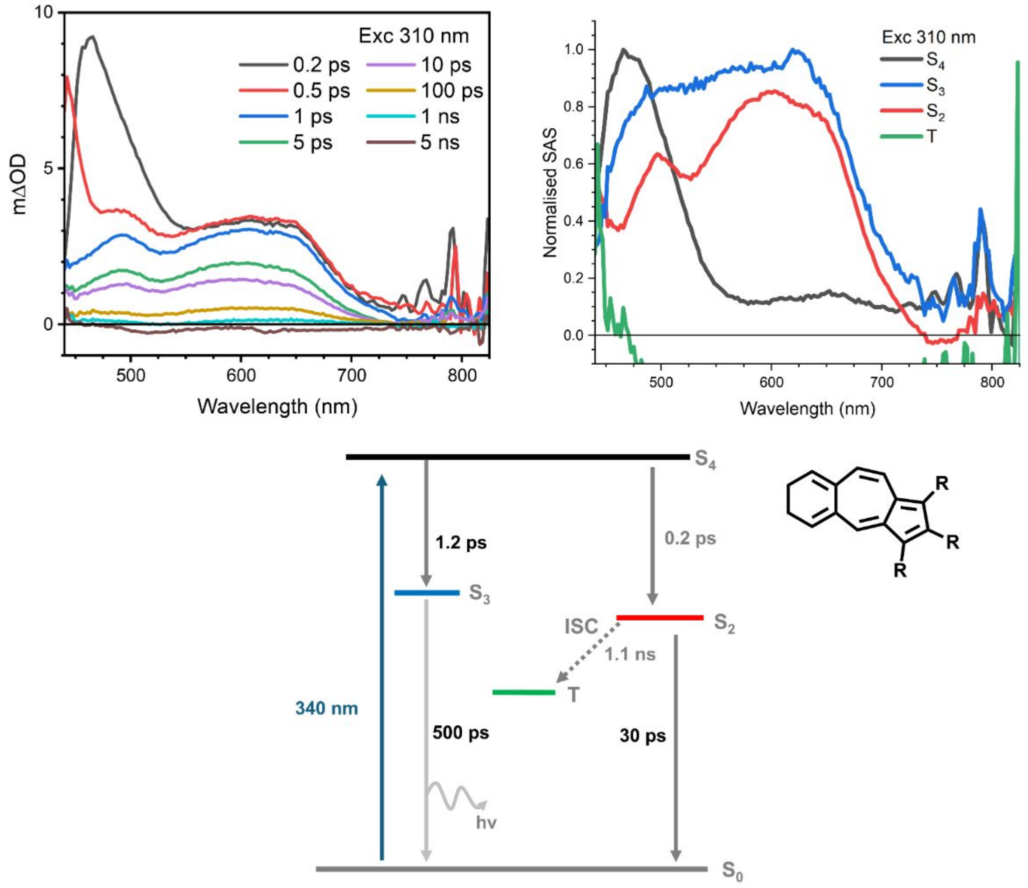

Figure S10. Ps-TAS characterization of **3Azu** upon excitation at (**a**) 310 nm. Excitation power has been maintained at 0.25 mW. (**b**) Result of the global analysis on the ps-TAS data showing the different species obtained from the data. (**c**) Jablonski diagram indicating the excited state dynamics of **3Azu** upon excitation.

The analysis of **3Azu** was complicated as excitation of **3Azu** at 400 nm gave negligible signal. The excitation of **3Azu** at 310 nm presents a large band 460 nm that quickly decays into a multiband with maxima at 490, 580 and 645 nm which evolves in an uneven form along the experiment timescale. To unravel the dynamics of the different species involved upon photoexcitation we performed global analysis on the data. The global analysis showed the presence of four different species (Supplementary **Fig. S8b**). According to the dynamics obtained by the global analysis we hypothesized the Jablonski diagram from Supplementary **Fig. S8c** where upon excitation to S_4 state, there is a formation of S_2 and S_3 in less than a picosecond. Each of these states would decay with a lifetime of 500 and 30 ps for S_3 and S_2, respectively. Also, the S_2 state is able to perform intersystem crossing to form triplets.

5Azu

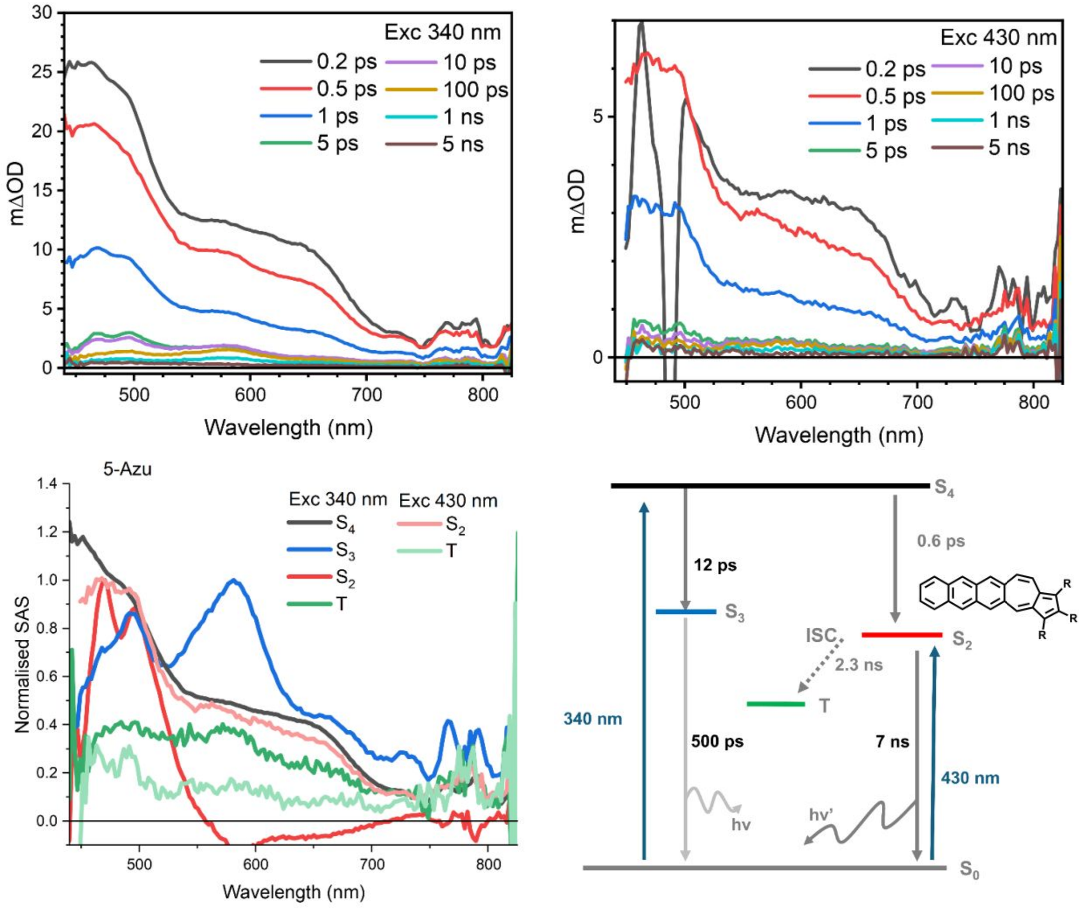

Figure S11. Ps-TAS characterization of **5Azu** upon excitation at (**a**) 340 and (**b**) 430 nm. Excitation power has been maintained at 0.25 mW. (**c**) Result of the global analysis on the ps-TAS data showing the different species obtained from the data. (**d**) Jablonski diagram indicating the excited state dynamics of **5Azu** upon excitation.

5Azu was excited at 340 and 430 nm to characterize their excited state dynamics. Global analysis performed on the data upon excitation at 430 nm indicated the presence of two different species: a band with two peaks at 465 and 490 nm with a tail that extends to the near infrared, and a featureless band that extends into the ultraviolet. This last species remains constant along the timescale of the technique, suggesting that this species to be a triplet state. Therefore, the state where it comes from must be a singlet state, namely S_2. The excitation at larger energy (340 nm) generated two new species according to the global analysis. A large intensity species that extends to the UV which decays in few picoseconds assigned to the larger energy singlet state, namely S_4. Upon formation, this S_4 state decays into S_2 and a new species with a big wide band at 590 nm, which we assign to S_3. This S_3 state decays with a lifetime of 500 ps in line with the PL lifetime of 670 ps.

VII. Theoretical Calculations

Theoretical calculations were carried out using Gaussian 09 software.[4] The ground-state structures were optimized by density functional theory (DFT) at B3LYP/6-31G* level.[5] The electrostatic potentials and FMO distributions were visualized using Gaussview 5.0 software. The charge-transfer integrals (*t*) were calculated at B3LYP/6-31G* level based on the single crystal structures.[6]

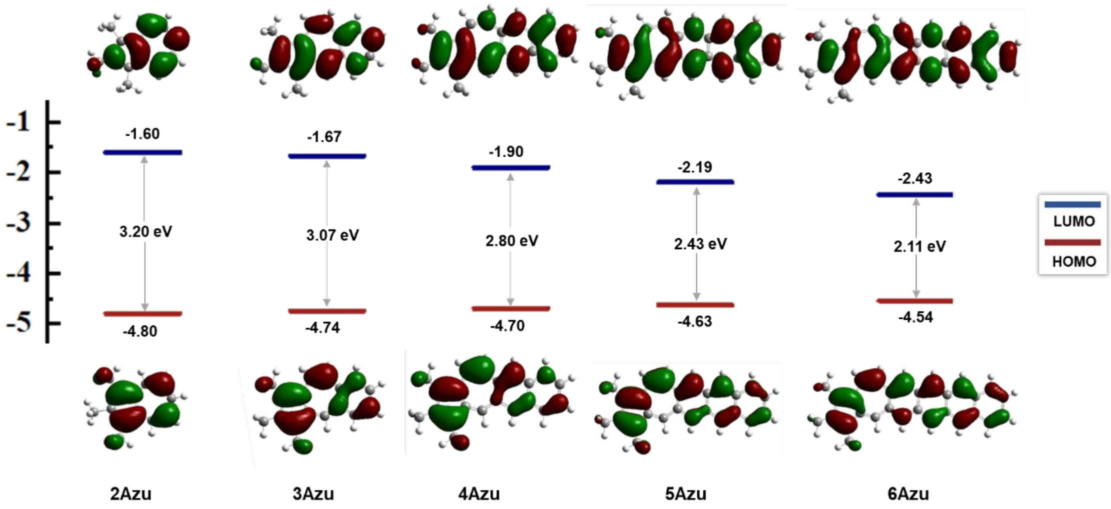

Figure S12. Calculated FMO distributions and orbital energy levels of **nAzus** (n = 2-6).

Table S5. Calculated orbital energy levels of **nAzus** and **nAcenes** (n = 2-6).

n	nAzu		nAcene	
	HOMO (eV)	LUMO (eV)	HOMO (eV)	LUMO (eV)
**2**	-4.80	-1.60	-5.79	-0.96
**3**	-4.74	-1.67	-5.23	-1.63
**4**	-4.70	-1.90	-4.86	-2.08
**5**	-4.63	-2.19	-4.6	-2.39
**6**	-4.54	-2.43	-4.41	-2.61

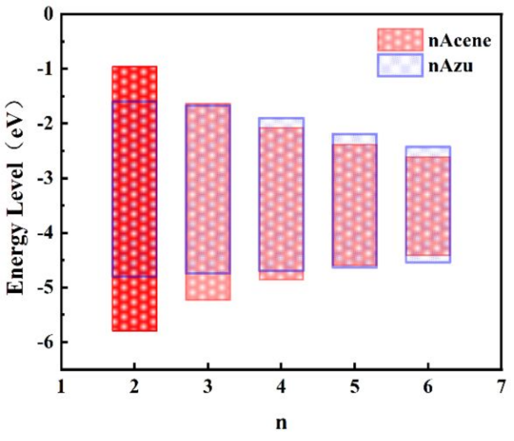

Figure S13. Calculated orbital energy levels of **nAzu**s and **nAcene**s (n = 2-6).

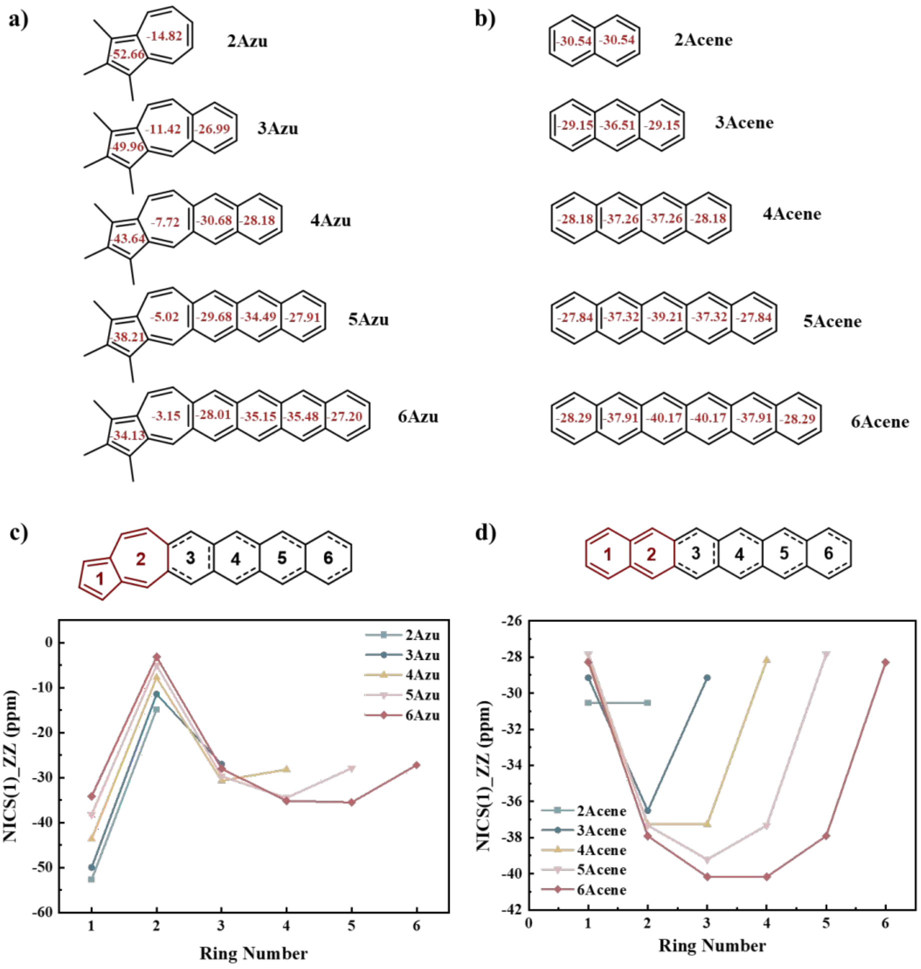

Figure S14. Calculated nucleus-independent chemical shift (NICS) of (**a**,**c**) **nAzu**s and (**b**,**d**) **nAcene**s (n = 2-6).

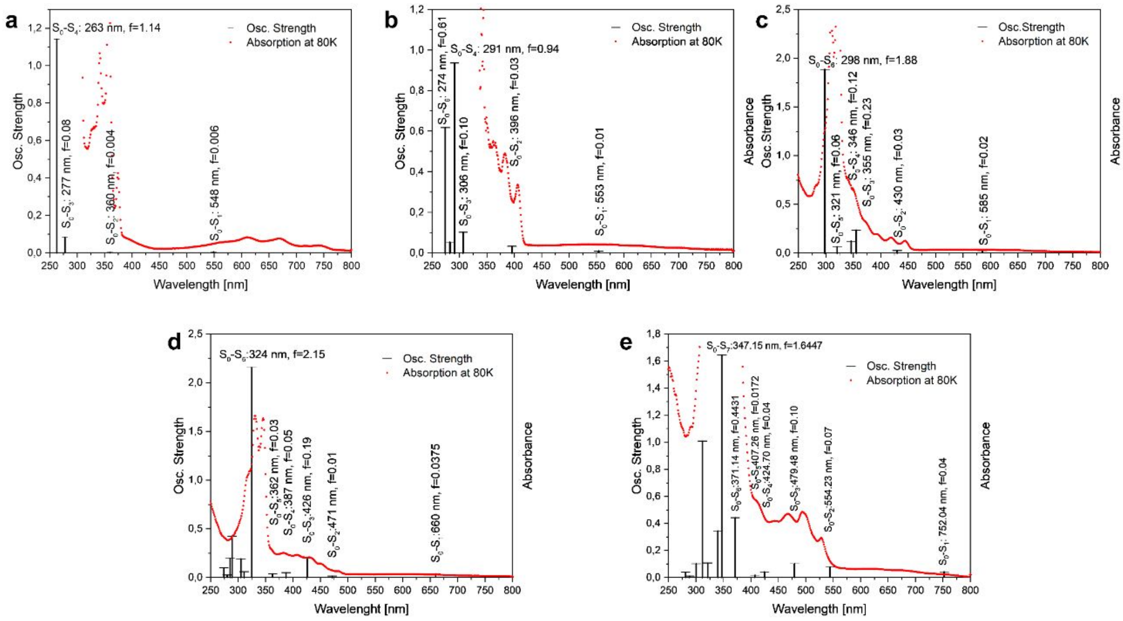

Figure S15. Calculated oscillator strength compared to experimental electronic absorption spectra at 80 K for **nAzu**s (**a,** n= 2; **b,** n= 3; **c,** n=4; **d,** n= 5; **e,** n=6) in 2-MeTHF. Calculations have been performed using TD-DFT at B3LYP/6-31G** level of theory.

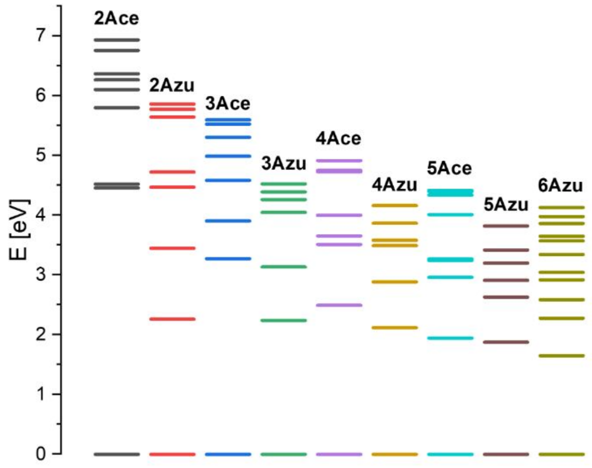

Figure S16. Calculated singlet energy levels of **nAzu**s and **nAce**s. Calculations have been performed using TD-DFT at B3LYP/6-31G** level of theory.

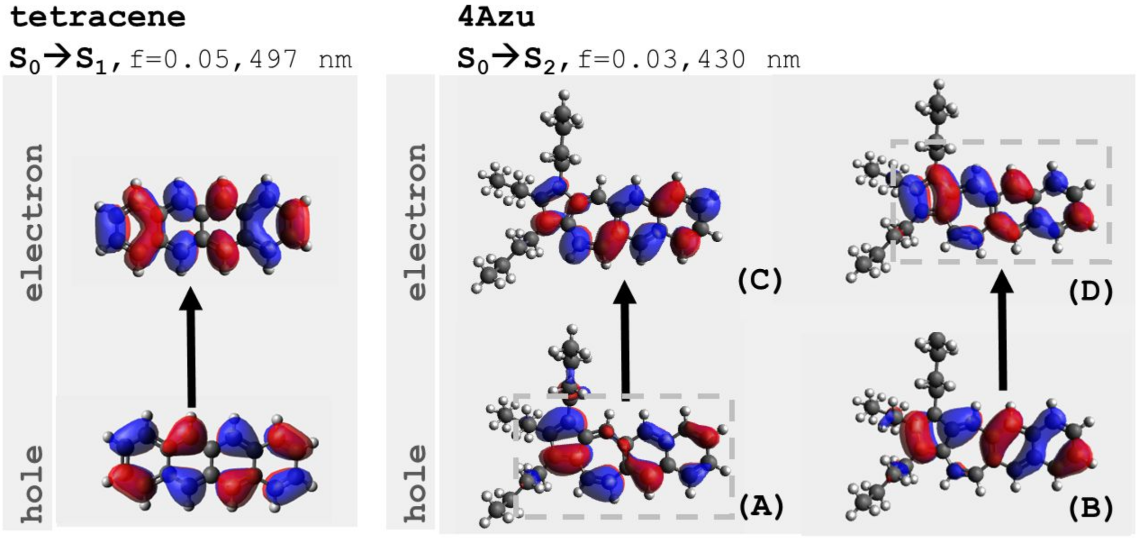

Figure S17. Natural transition orbitals for the $S_0 \rightarrow S_1$ excitation calculated at 497 nm with oscillator strength of 0.05 for tetracene and for the $S_0 \rightarrow S_2$ at 430 nm with oscillator strength of 0.03 for **4Azu** from TD-DFT/B3LYP/6-31G** calculations.

VIII. OFET Device Fabrication

Device fabrication of single crystal OFETs.

The surface of the substrates with 300-nm-thick thermally oxidized SiO_2 on doped Si was cleaned orderly with deionized water, piranha solution (H_2SO_4/H_2O_2 = 7:3), deionized water, isopropyl alcohol, and finally were blown dry with high-purity nitrogen gas. Octadecyltrichlorosilane (OTS) modifying SiO_2/Si wafers was carried out with the vapor-deposition method: the cleaned wafers were dried under vacuum at 90 °C for 0.5 h to eliminate the moisture. When the temperature decreased to 70 °C, a small drop of OTS was dropped onto the wafers. Subsequently, this system was heated to 120 °C for 2 h under vacuum. OTS modified SiO_2/Si wafers used here were cleaned with *n*-hexane, chloroform, and isopropyl alcohol in sequence, and finally were blown dry with high-purity nitrogen gas. The organic crystal was prepared on the OTS-treated substrates by a drop-casting process, gold source and drain contacts (60 nm in thickness) were deposited on the organic layer by vacuum evaporation, affording a bottom-gate top-contact configuration.

Transfer and output characteristics of OFETs were collected using a semiconductor parameter analyzer (Keithley BT1500A). Field effect mobility values (μ_{sat}) were estimated from the saturation regime using the following equation:

$$-I_D = (WC_i/2L)\ \mu_{sat}\ (V_G - V_{th})^2$$

C_i is the capacitance of gate insulator, V_{th} is the threshold voltage, and L and W are length and width of the channel, respectively.

Table S6. OFET device performance of **5Azu**[a]

Compound	μ ($cm^2\ V^{-1}\ s^{-1}$) max (avg)	V_{th} (V) min (avg)	$I_{on/off}$ max (avg)
**5Azu**	0.10 (0.04)	-10 (-23)	10^5 (10^5)

[a]Averaged from 5 devices; all devices were tested under N_2 atmosphere.

IX. References

[1] L. Zhao, R. I. Kaiser, W. Lu, M. Ahmed, M. M. Evseev, E. K. Bashkirov, V. N. Azyazov, C. Tönshoff, F. Reicherter, H. F. Bettinger, A. M. Mebel, *Angew. Chem. Int. Ed.* **2020**, *59*, 11334-11338.

[2] D. Kato, H. Sakai, N. V. Tkachenko, T. Hasobe. *Angew. Chem. Int. Ed.* **2016**, *55*, 5230-5234.

[3] L. Huang, M. Rudolph, F. Rominger, A. S. K. Hashmi, *Angew. Chem. Int. Ed.* **2016**, *55*, 4808-4813.

[4] M. J. Frisch, G. W. Trucks, H. B. Schlegel, G. E. Scuseria, M. A. Robb, J. R. Cheeseman, G. Scalmani, V. Barone, B. Mennucci, G. A. Petersson, H. Nakatsuji, M. Caricato, X. Li, H. P. Hratchian, A. F. Izmaylov, J. Bloino, G. Zheng, J. L. Sonnenberg, M. Hada, M. Ehara, K. Toyota, R. Fukuda, J. Hasegawa, M. Ishida, T. Nakajima, Y. Honda, O. Kitao, H. Nakai, T. Vreven, J. A. Jr. Montgomery, J. E. Peralta, F. Ogliaro, M. Bearpark, J. J. Heyd, E. Brothers, K. N. Kudin, V. N. Staroverov, R. Kobayashi, J. Normand, K. Raghavachari, A. Rendell, J. C. Burant, S. S. Iyengar, J. Tomasi, M. Cossi, N. Rega, N. J. Millam, M. Klene, J. E. Knox, J. B. Cross, V. Bakken, C. Adamo, J. Jaramillo, R. Gomperts, R. E. Stratmann, O. Yazyev, A. J. Austin, R. Cammi, C. Pomelli, J. W. Ochterski, R. L. Martin, K. Morokuma, V. G. Zakrzewski, G. A. Voth, P. Salvador, J. J. Dannenberg, S. Dapprich, A. D. Daniels, Ö. Farkas, J. B. Foresman, J. V. Ortiz, J. Cioslowski, D. J. Fox, Gaussian 09, Revision D.01, Gaussian, Inc., Wallingford CT, **2009**.

[5] A. D. Becke, *J. Chem. Phys.* **1993**, *98*, 5648-5652.

[6] T. Lu, F. Chen, Multiwfn: A Multifunctional Wavefunction Analyzer, *J. Comput. Chem.* **2012**, 33, 580-592.

X. Copies of ^1H and ^{13}C NMR Spectra

^1H NMR spectrum of **2c** in $CDCl_3$

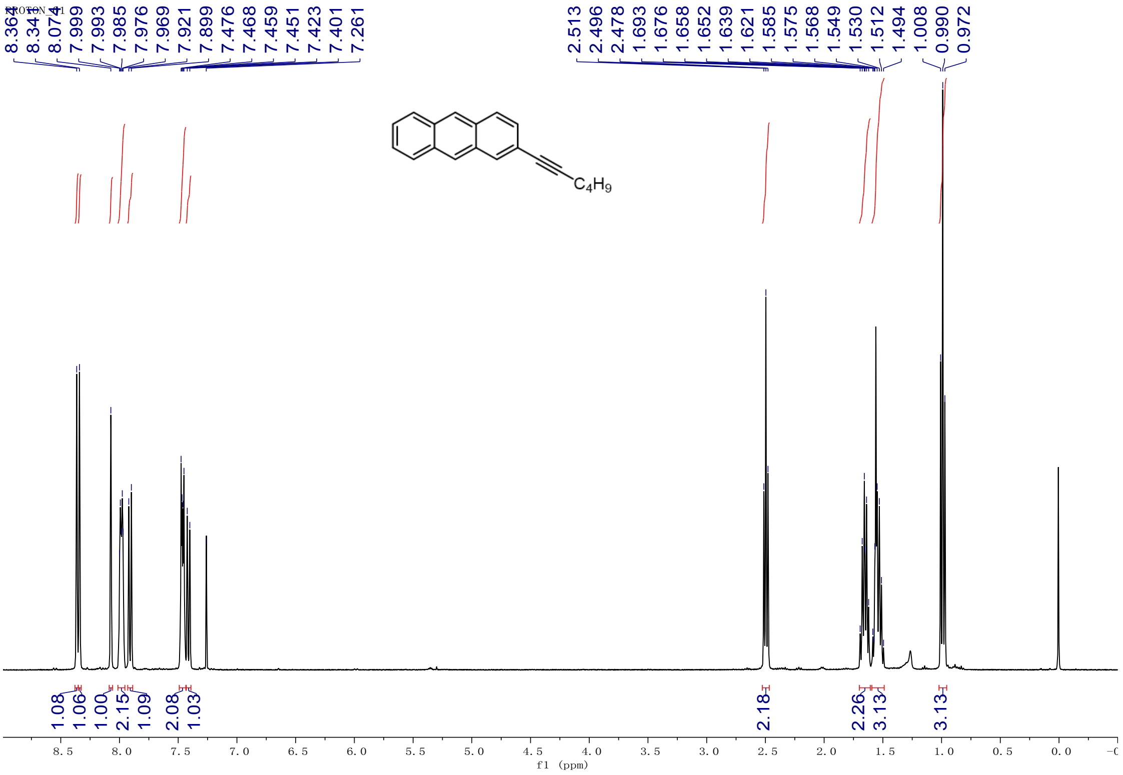

^{13}C NMR spectrum of **2c** in $CDCl_3$

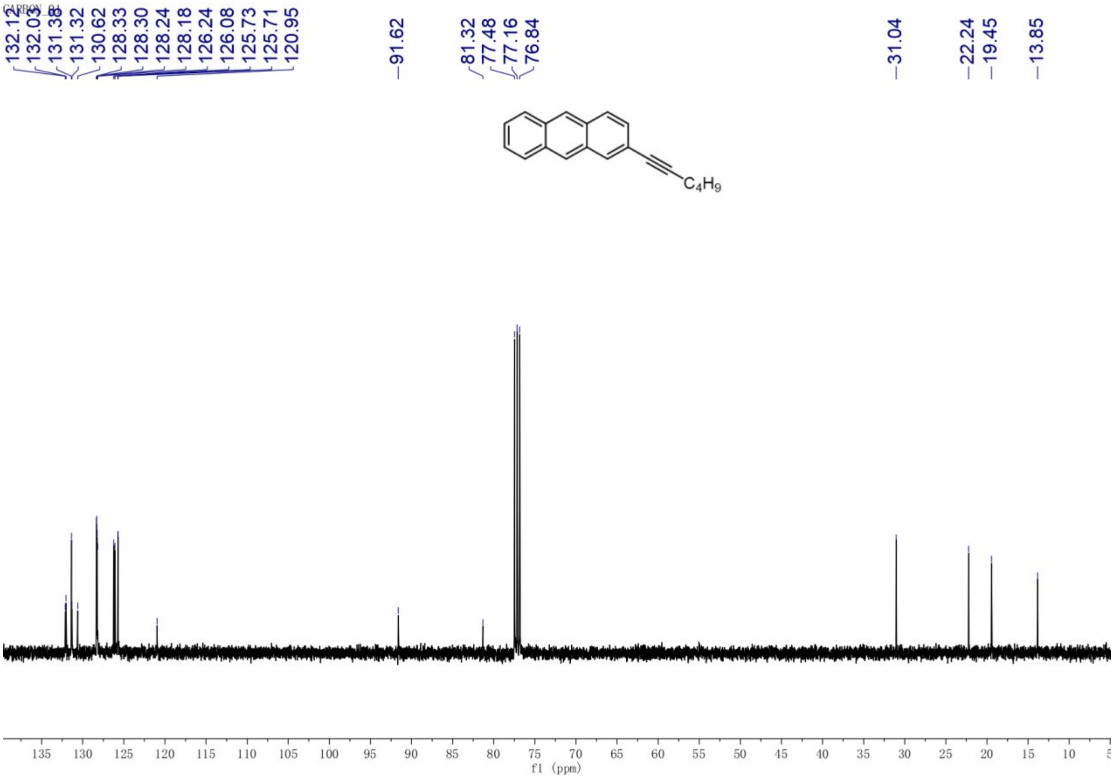

^1H NMR spectrum of **2d** in $CDCl_3$

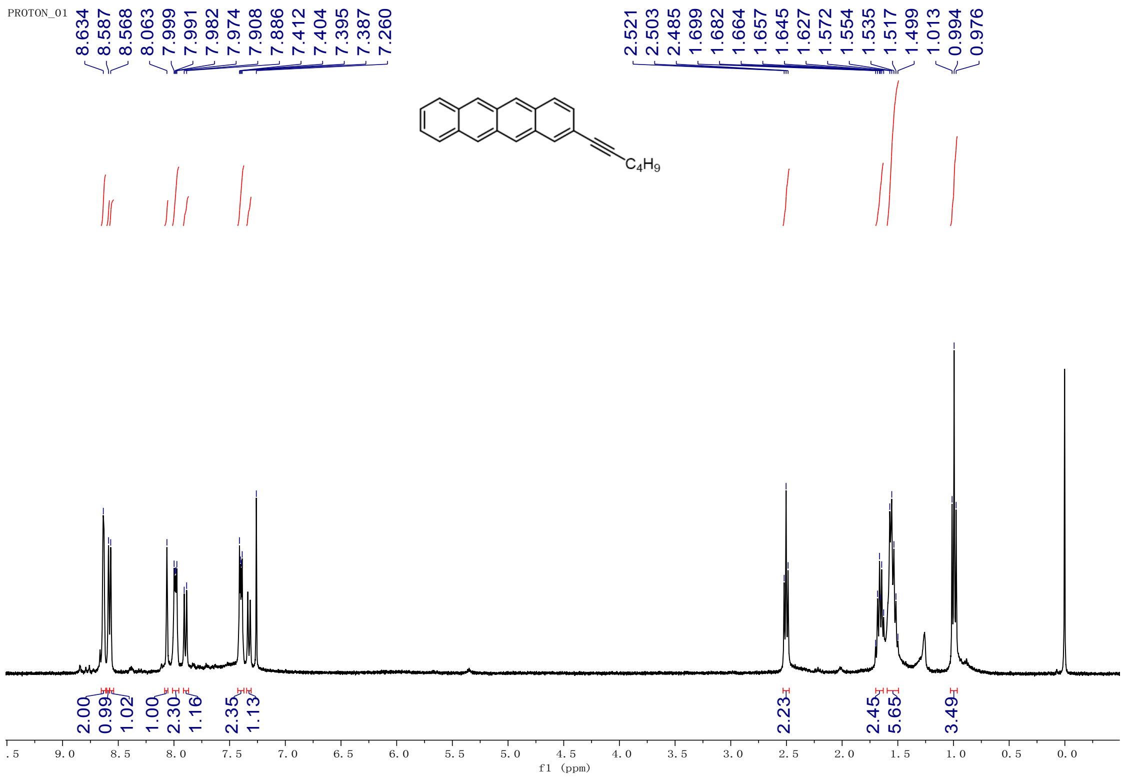

^{13}C NMR spectrum of **2d** in $CDCl_3$

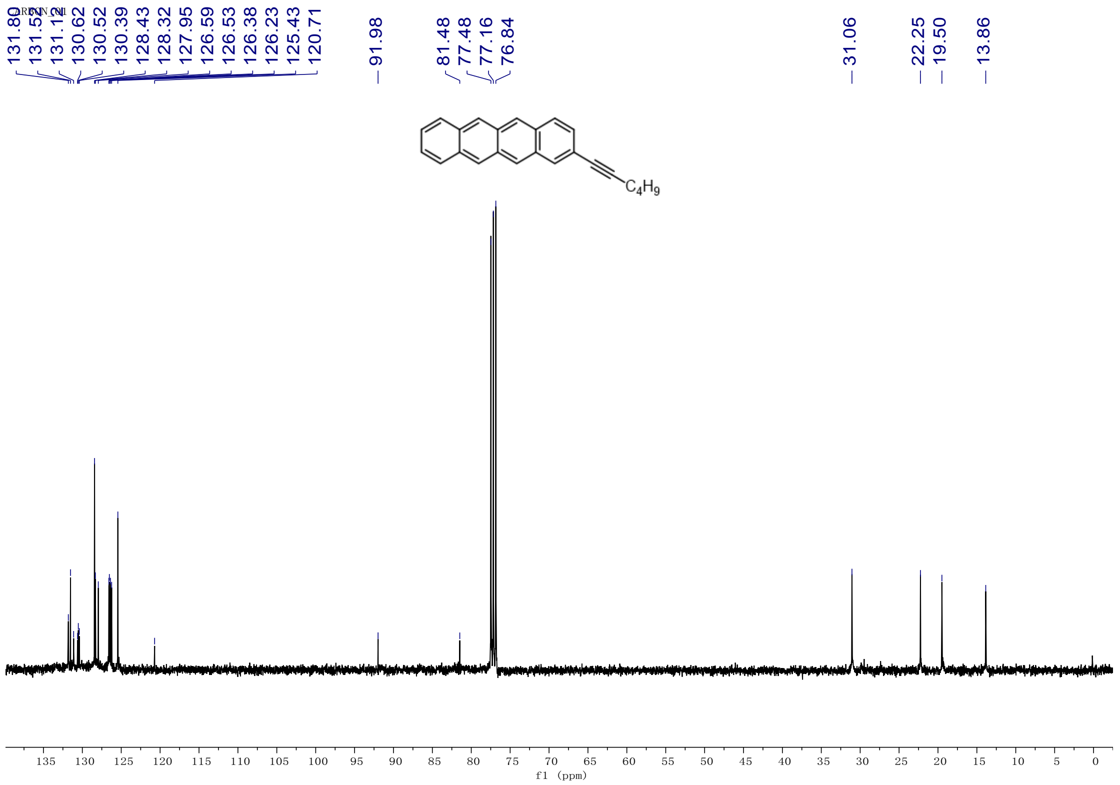

^{1}H NMR spectrum of **2e** in $CDCl_3$

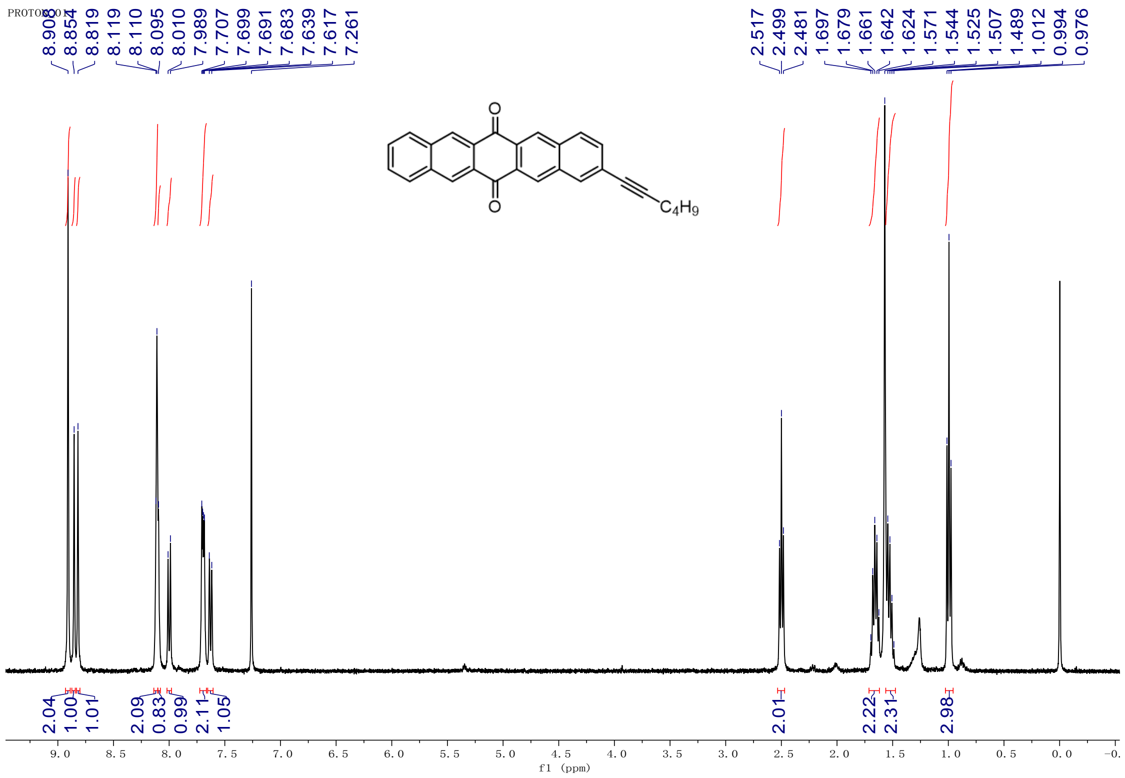

^{13}C NMR spectrum of **2e** in $CDCl_3$

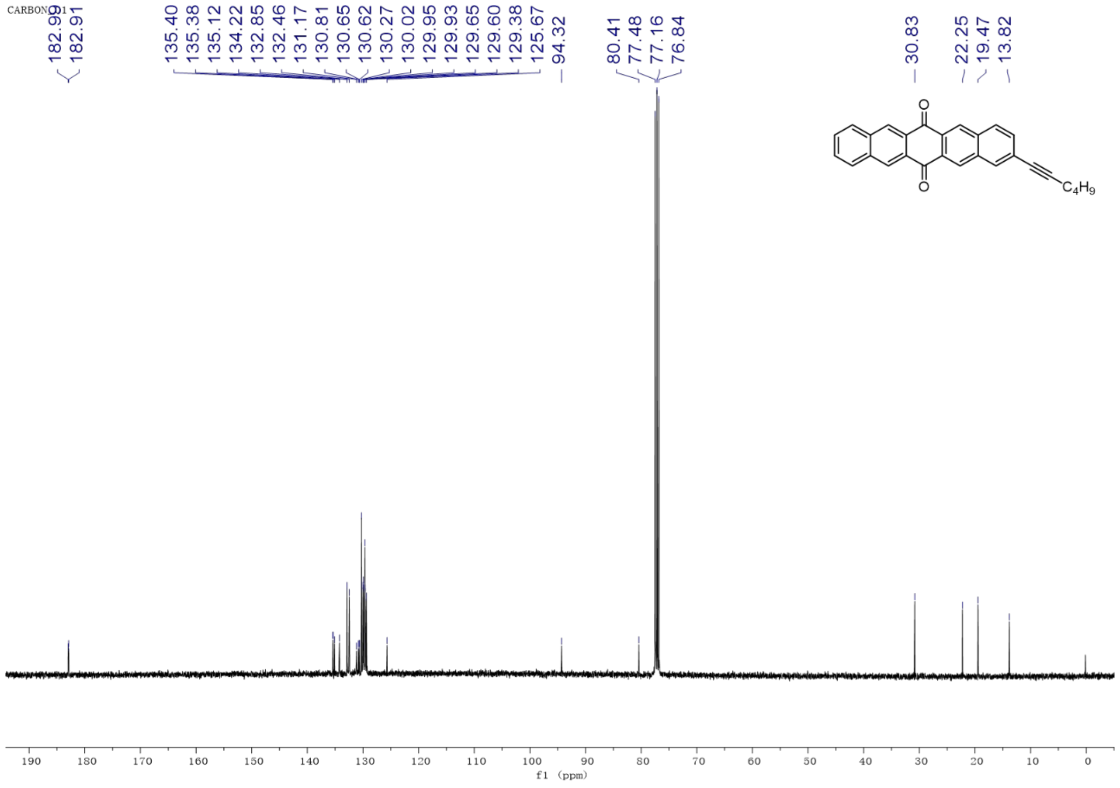

^{1}H NMR spectrum of **2Azu** in $CDCl_3$

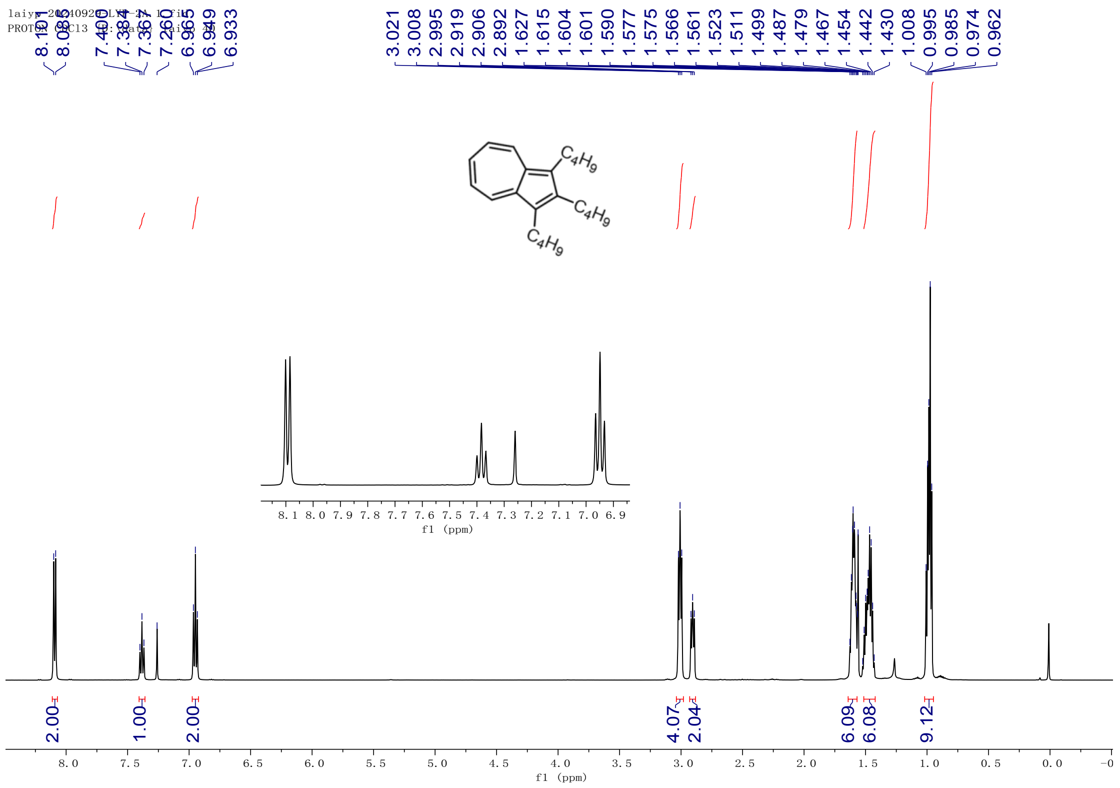

^{13}C NMR spectrum of **2Azu** in $CDCl_3$

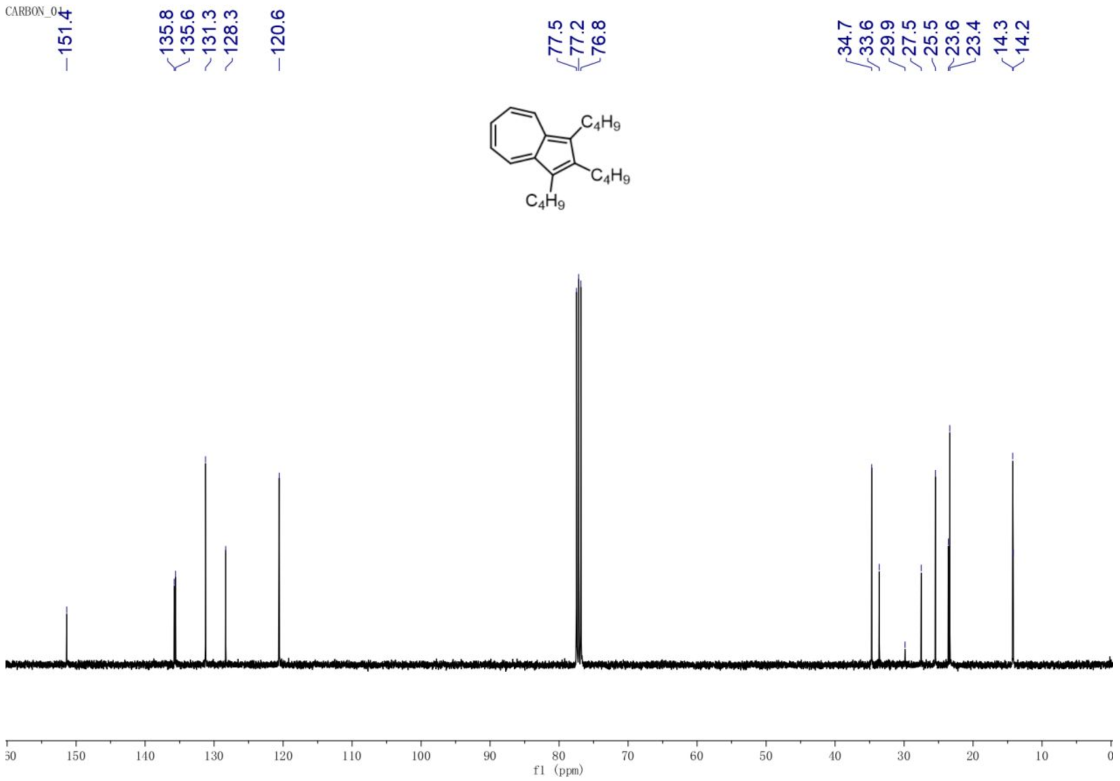

^{1}H NMR spectrum of **3Azu** in $CDCl_3$

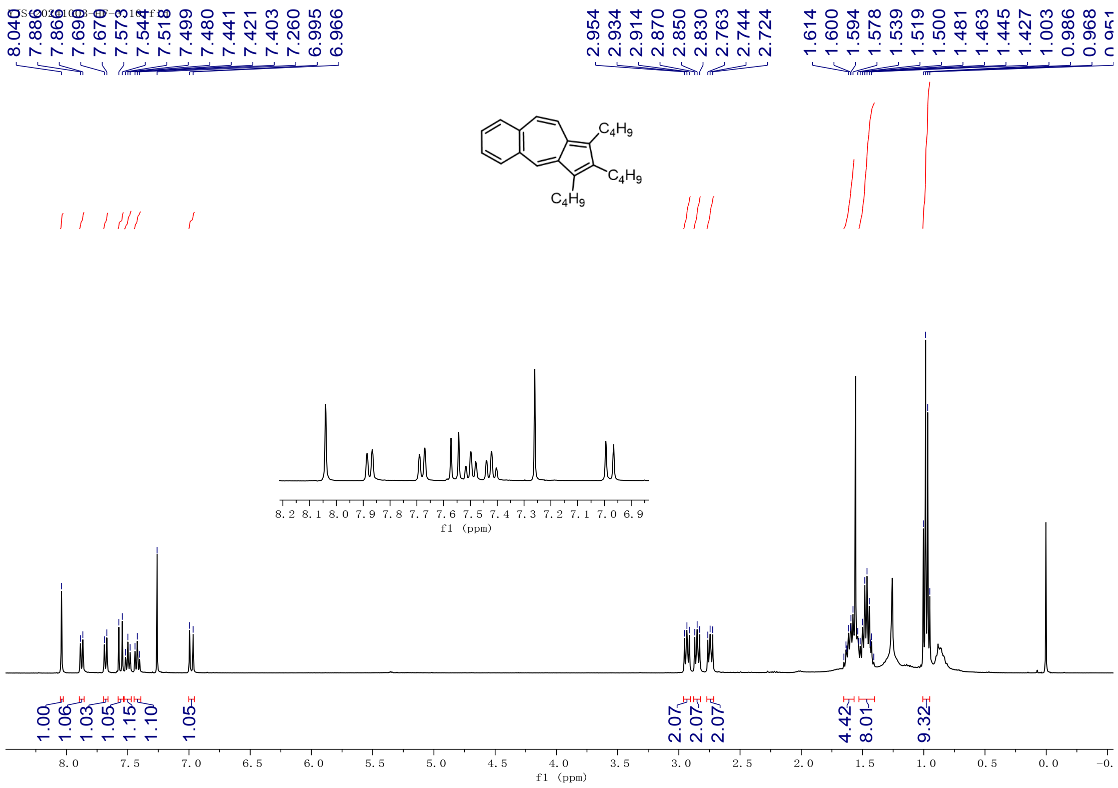

^{13}C NMR spectrum of **3Azu** in $CDCl_3$

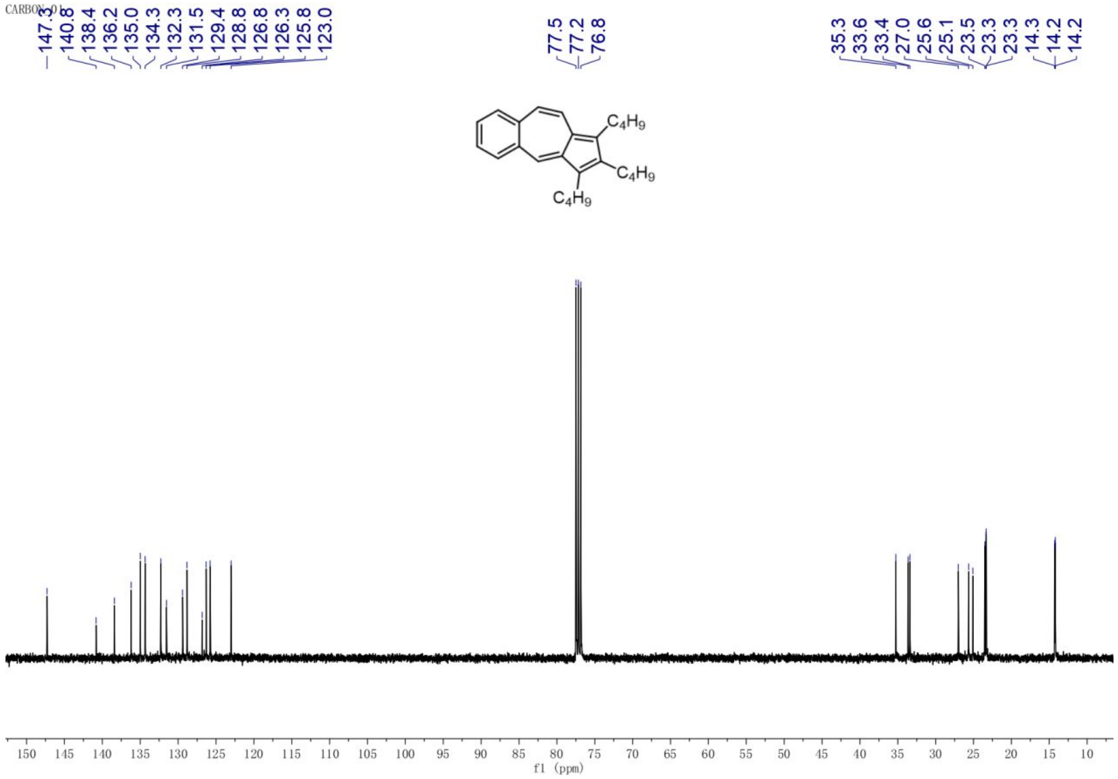

^{1}H NMR spectrum of **4Azu** in $CDCl_3$

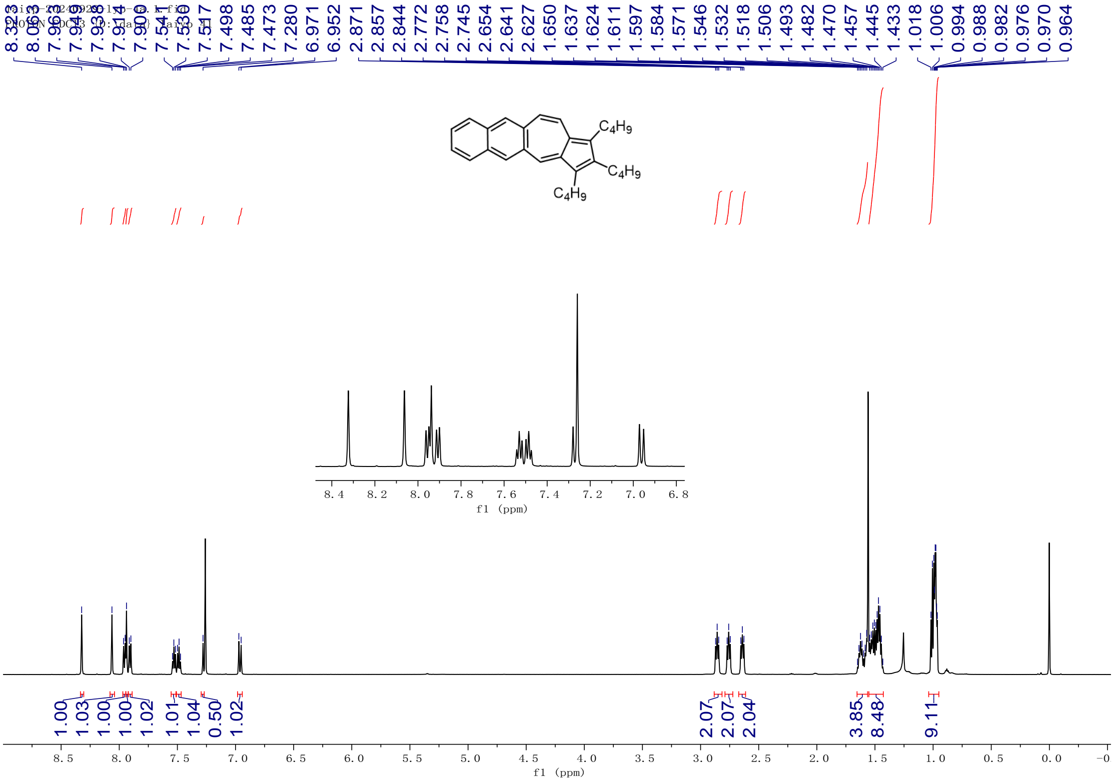

^{13}C NMR spectrum of **4Azu** in $CDCl_3$

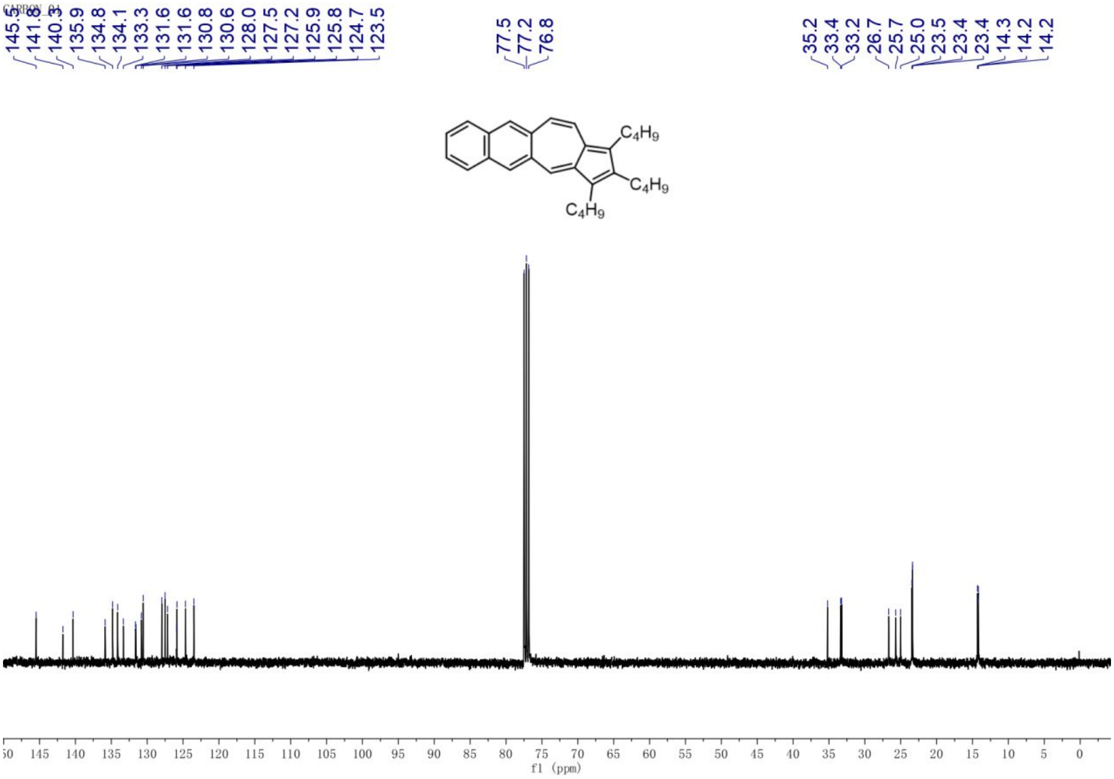

^{1}H NMR spectrum of **5Azu** in $CDCl_3$

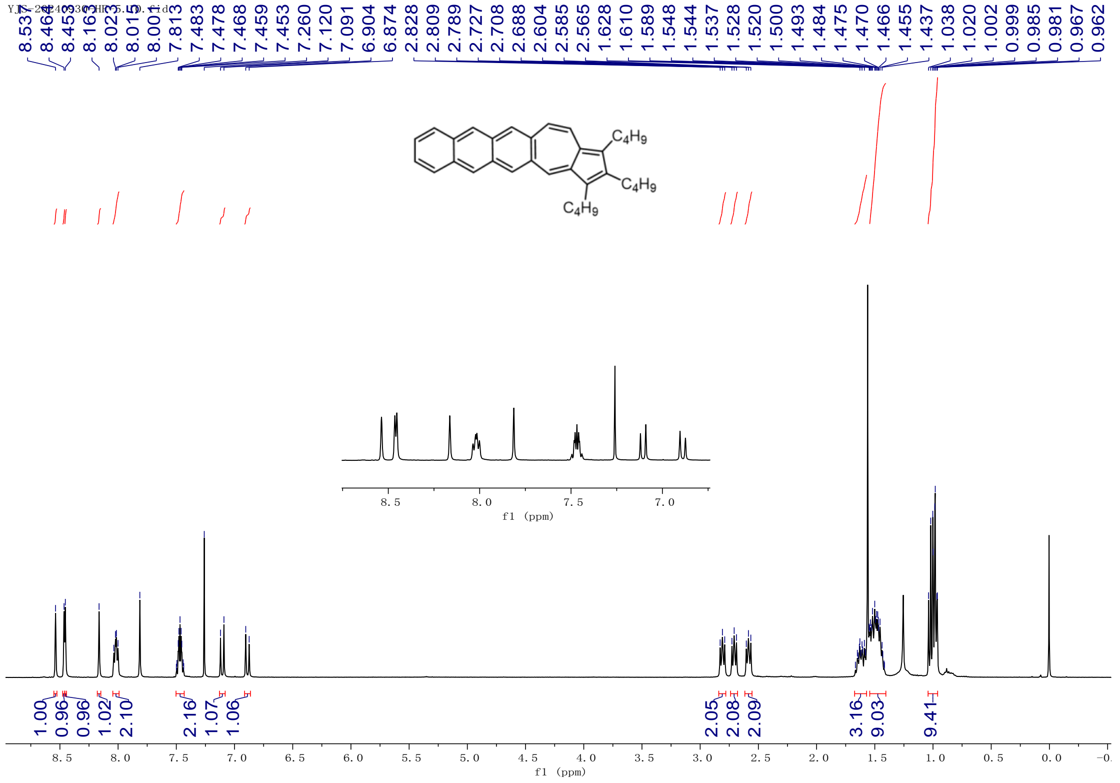

^{13}C NMR spectrum of **5Azu** in $CDCl_3$

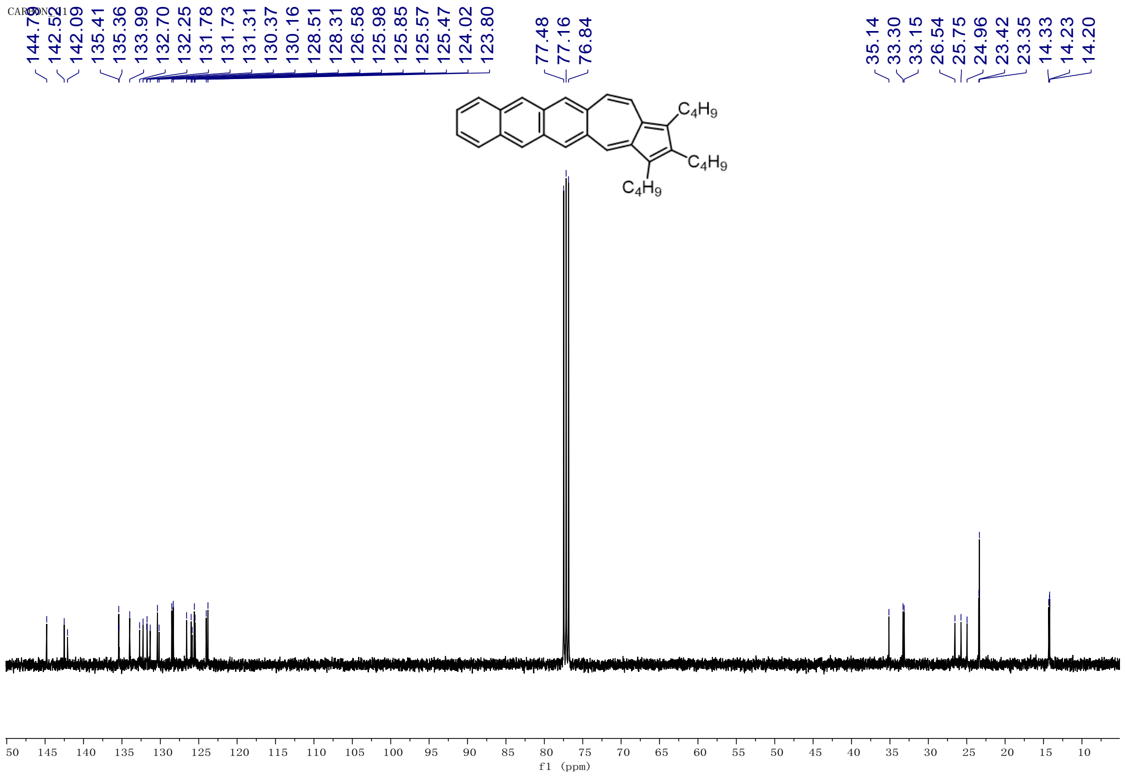

^{1}H NMR spectrum of **6Azu-pr** in $CDCl_3$

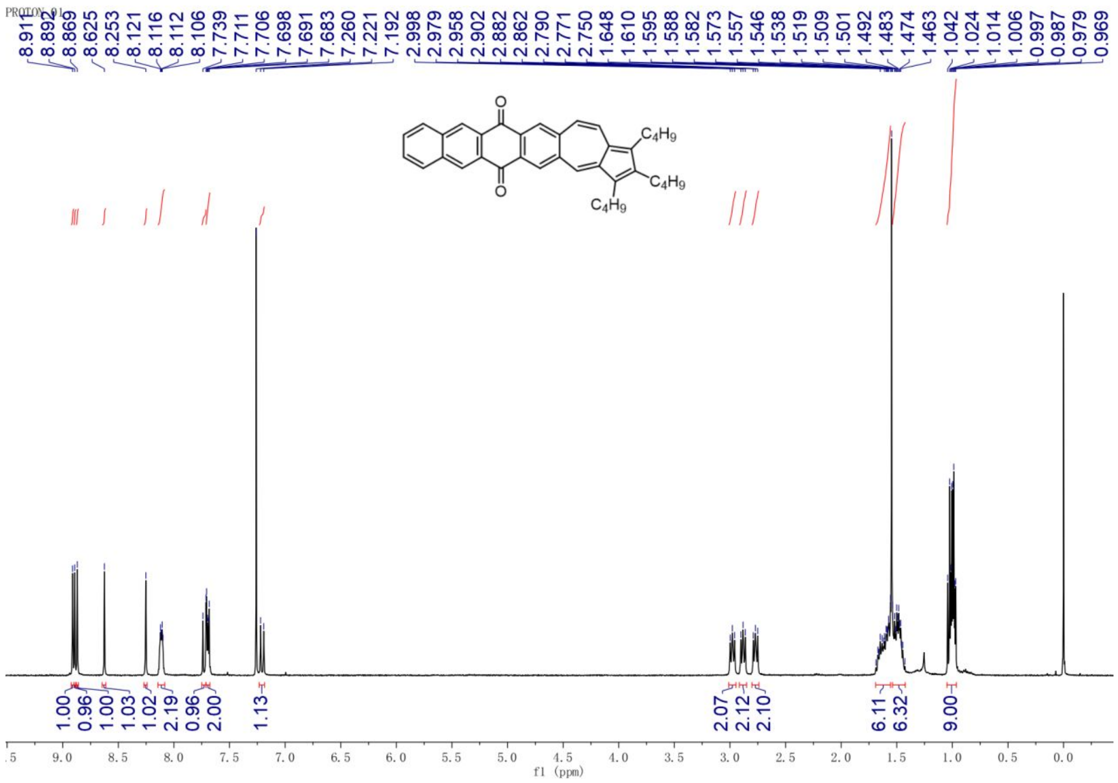

^{13}C NMR spectrum of **6Azu-pr** in $CDCl_3$

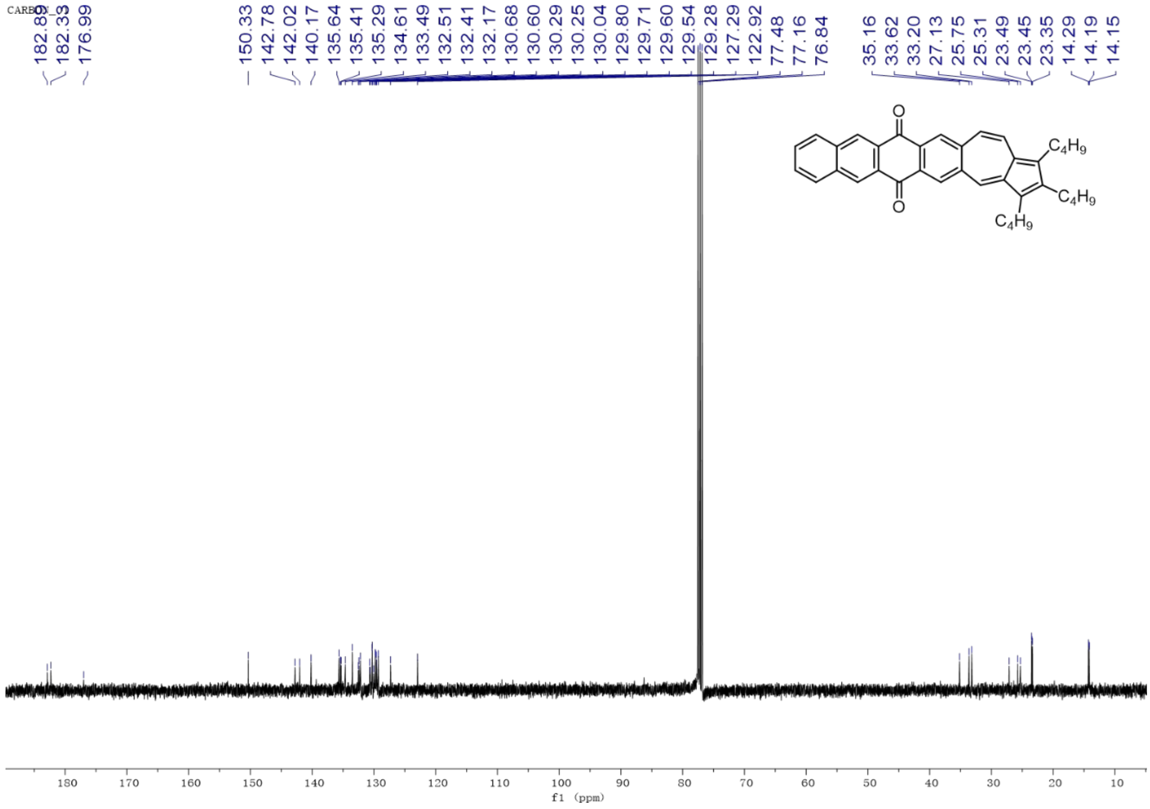

^{1}H NMR spectrum of **6Azu** in $CDCl_3$

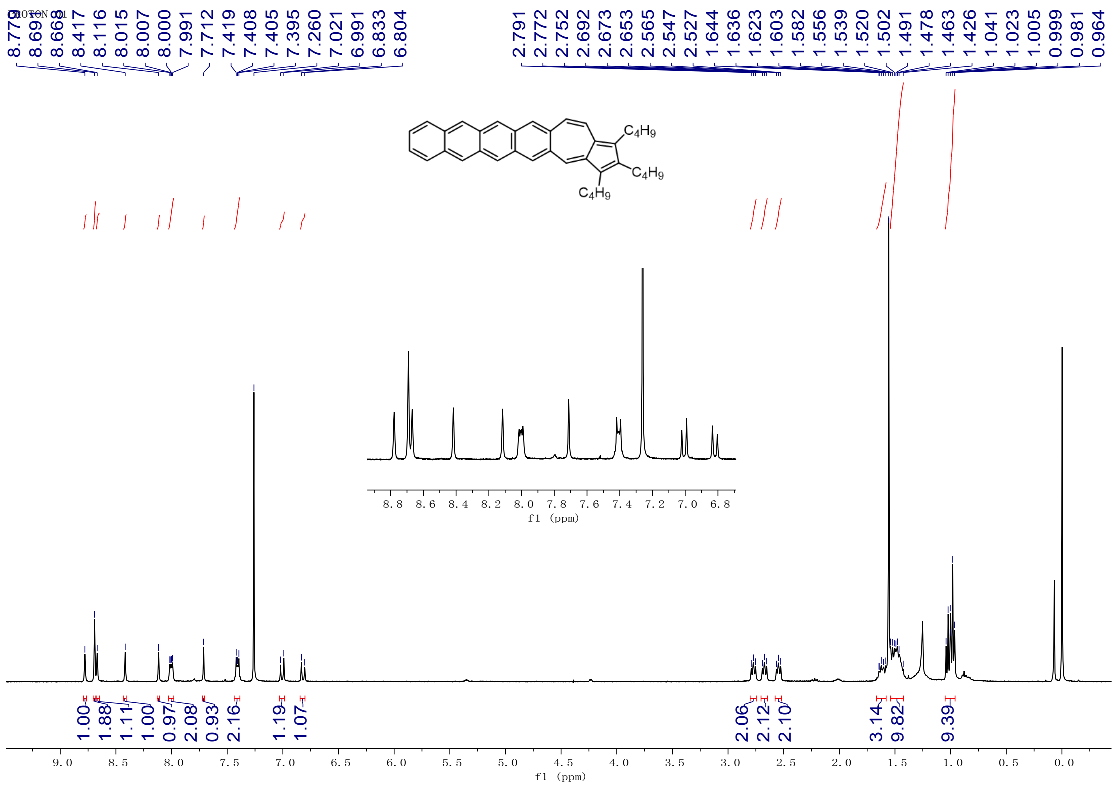

^{13}C NMR spectrum of **6Azu** in Tetrachloroethane-d_2

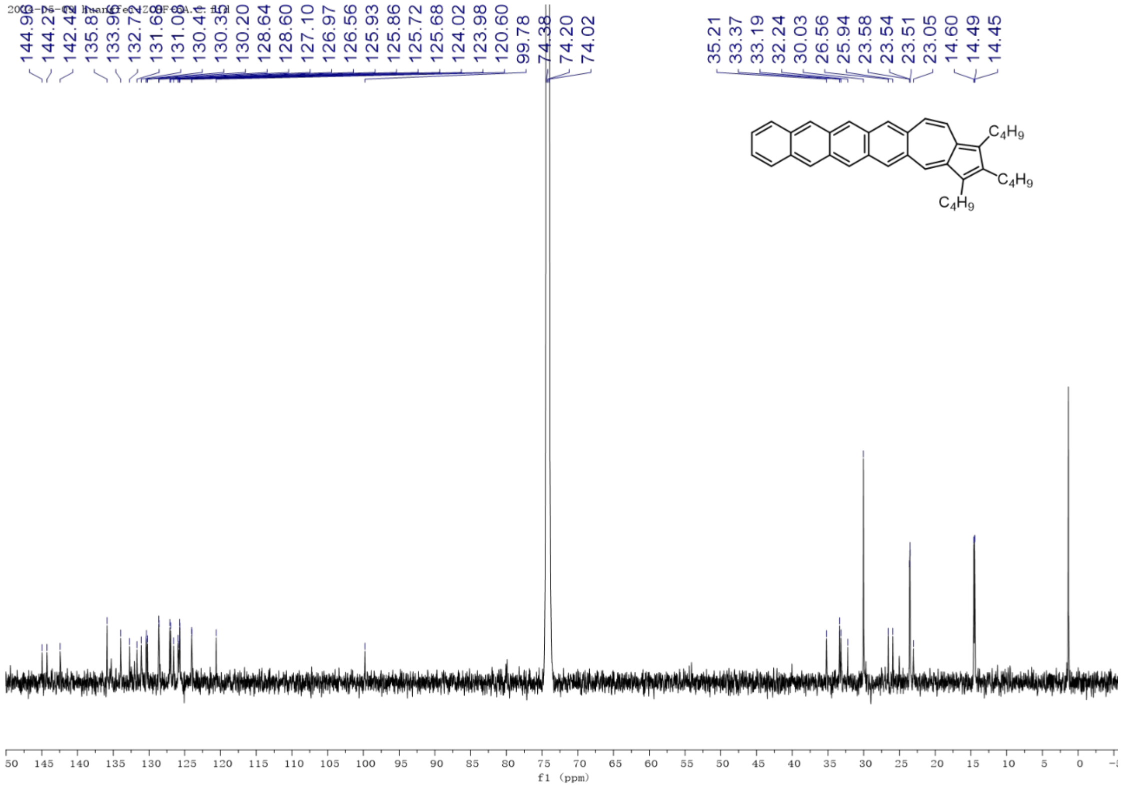

Supplement: Supplementary file 1 — ja4c11186_si_001.pdf [file ja4c11186_si_001.pdf]
